# Integration of Serum and Liver Metabolomics with Antioxidant Biomarkers Elucidates Dietary Energy Modulation of the Fatty Acid Profile in Donkey Meat

**DOI:** 10.3390/antiox15010140

**Published:** 2026-01-21

**Authors:** Li Li, Yanli Zhao, Yongmei Guo, Binlin Shi, Jing Zhang, Fanzhu Meng, Fang Hui, Qingyue Zhang, Xiaoyu Guo, Sumei Yan

**Affiliations:** Key Laboratory of Animal Nutrition and Feed Science, Universities of Inner Mongolia Autonomous Region, Hohhot 010018, China; lily972021@emails.imau.edu.cn (L.L.); ylzhao2017@imau.edu.cn (Y.Z.); ymguo2020@imau.edu.cn (Y.G.); shibl@imau.edu.cn (B.S.); alicezqy@emails.imau.edu.cn (J.Z.); fanzhumeng@emails.imau.edu.cn (F.M.); hf2021@emails.imau.edu.cn (F.H.); zhangqy2024@imau.edu.cn (Q.Z.)

**Keywords:** meat donkey, dietary energy, fatty acids, serum and liver metabolome, oxidative stress

## Abstract

Donkey meat is valued for its high protein, unsaturated fats, and low cholesterol. Fatty acid (FA) composition critically influences meat quality and is modulated by dietary energy levels. Twenty-four meat donkeys (male) were randomly divided into three groups: a low-energy group (LEG), a medium-energy group (MEG), and a high-energy group (HEG). The trial lasted for 135 days, with dietary digestible energy levels adjusted during the pre-fattening, mid-fattening, and late-fattening phases according to the experimental design. The results showed that MEG and HEG interventions significantly upregulated tissue polyunsaturated fatty acid (PUFA) and n-3 PUFA content while reducing n-6/n-3 ratios, concomitant with enhanced activity and gene expression of most lipid-metabolizing enzymes. Notably, MEG further elevated antioxidant enzyme activities and anti-inflammatory mediators while suppressing pro-inflammatory factors. MEG and HEG significantly upregulated serum cholestane-3,7,12,25-tetrol-3-glucuronide and cortisol, along with hepatic choline, lysoPC(20:2(11Z,14Z)), glycocholic acid, and cholestane-3,7,12,25-tetrol-3-glucuronide. These modified metabolites were predominantly enriched in key metabolic pathways: pentose and glucuronate interconversions, primary bile acid biosynthesis, steroid hormone biosynthesis, glycerophospholipid metabolism, purine metabolism, and glutathione metabolism. Additionally, compared to HEG, MEG improved the antioxidant activities and immune signaling molecule levels with elevated pyroglutamic acid, glutathione, choline, inosine, adenine, and uric acid. Thus, moderately elevated dietary energy levels may enhance FA profiles in muscular and adipose tissues through coordinated regulation of lipid-metabolizing enzymes and associated gene expression, with serum and hepatic metabolites actively participating in these regulatory pathways. However, excessive energy intake could induce oxidative stress in donkeys.

## 1. Introduction

With the improvement in living standards and health consciousness, people have higher needs for meat quality. Donkey meat is gaining popularity worldwide due to its desirable nutritional properties, including high protein content, low fat, and a favorable fatty acid (FA) profile rich in unsaturated fatty acids (UFAs) [1]. Meat’s nutritional value, sensory qualities, and potential health effects are all greatly influenced by its FA profile, particularly the ratio of unsaturated fatty acids (UFAs) to saturated fatty acids (SFAs) [2]. Research has confirmed that dietary consumption of n-3 polyunsaturated fatty acids (PUFAs) has favorable effects on human physiology, including the modulation of blood pressure, immunological responses, and cardiovascular function [3]. Both n-6 and n-3 PUFA are vital for human nutrition, and preserving the proper n-6/n-3 ratio in the diet is necessary for promoting ideal physiological equilibrium. Additionally, it has been demonstrated that docosahexaenoic acid (DHA) and eicosapentaenoic acid (EPA) affect the activity of membrane-associated proteins, including immunogenic receptors and signaling molecules [4], with DHA also taking part in anti-inflammatory signaling pathways [5]. To increase fat quality, it is crucial to optimize the FA content of donkey meat and adipose tissue.

In the longissimus dorsi muscle of fattening yaks, high dietary energy intake was shown to increase intramuscular fat deposition while also increasing the proportion of PUFAs and decreasing that of SFAs in the intramuscular lipid fraction [6]. Similarly, Yang et al. [7] observed elevated serum levels of C14:0, C16:1, C18:2n6c, and n-3 PUFAs (e.g., C20:5n3 and C22:6n3) in high-energy-fed yaks. Lowering the metabolizable energy in finishing pigs’ diet from 13.82 to 13.40 MJ/kg caused a spike in saturated fatty acids while bringing down the levels of PUFAs in their meat [8]. Nonetheless, the fundamental processes need additional study.

The liver is vital for the synthesis and metabolism of FA within the organism, serving as a central hub for lipid homeostasis. Notably, hepatic lipogenesis peaks during active feeding periods, driven by insulin signaling and transcriptional regulators such as  Sterol Regulatory Element-Binding Protein (SREBP)-1c, while FA oxidation is modulated by the peroxisome proliferator-activated receptor (PPAR) and circadian clock genes to align with energy demands [9]. An altered energy supply may impair lipid homeostasis and contribute to metabolic disorders [10]. Dietary energy levels can affect the FA composition in the blood and liver, which may be responsible for the changes in FA in muscle and adipose tissue. The research team previously demonstrated that suitably raising the dietary energy can encourage the growth of meat donkeys [11] and enhance the deposition of nutrients in the muscles [12], which may be associated with alterations in cecal microorganisms and metabolites [13]. However, whether it affects the FA composition of body tissues has not yet been studied. We hypothesized that the dietary energy level could alter the fatty acid (FA) composition of the longissimus dorsi muscle and subcutaneous fat through modulating the metabolite profile in the liver and blood, as well as the activity of enzymes and the expression of genes involved in FA metabolism. To assess the effects of various energy levels on the amount of FA in donkey muscle and adipose tissue and to investigate potential mechanisms based on liver and serum metabolomes, associated enzyme activities, and gene expression, the current experiment was conducted. The results have important theoretical and practical ramifications for raising the nutritional content, quality, and profitability of donkey meat production.

## 2. Materials and Methods

### 2.1. Experimental Design, Diet, and Feeding Management

The experimental design was based on a completely randomized single-factor arrangement. Twenty-four young male donkeys, all approximately one year old and with comparable body weights ranging from 125 to 175 kg, were evenly distributed across three distinct dietary regimens: a low-energy-diet group (LEG), a medium-energy-diet group (MEG), and a high-energy-diet group (HEG). The research commenced with a 10-day adjustment period before proceeding to the 135-day main experiment. Throughout the trial, the subjects were fed specialized rations containing varying concentrations of metabolizable energy (measured on a dry weight basis): specifically, 12.08, 13.38, and 14.40 MJ/kg during the initial weight-gain phase (days 1–45); 13.01, 14.07, and 15.60 MJ/kg in the middle period of fattening (days 46–90); and 13.54, 14.93, and 16.23 MJ/kg by the final stretch of the fattening process (days 91–135). Twice each day at 7:00 AM and 2:00 PM, the donkeys received their rations. Each animal was housed in an individual pen with a separate feeding trough to eliminate social interactions and potential pen effects, with individual feeding stations and round-the-clock access to fresh water. The experimental protocol remained consistent across all groups throughout the study. Details regarding the dietary formulation and nutritional specifications can be found in Table 1 and Table S1, while the fatty acid composition is outlined in Table S2.

**Table 1:** Dietary nutrient level in fattening period (air-dry basis, %).

Index	1–45 d			46–90 d			91–135 d		
	LEG	MEG	HEG	LEG	MEG	HEG	LEG	MEG	HEG
Digestible energy, MJ/kg ^1^	12.08	13.38	14.40	13.01	14.27	15.60	13.54	14.93	16.23
Crude protein	14.53	15.06	15.06	13.02	13.04	13.17	12.48	12.67	12.72
Ether extract	5.69	6.43	7.13	6.06	6.50	6.97	6.47	6.95	7.32
Neutral detergent fiber	57.29	55.38	50.99	48.01	46.7	44.21	46.94	43.86	41.63
Acid detergent fiber	37.90	38.41	36.54	31.19	30.85	28.72	31.95	30.29	27.02
Calcium	1.33	1.38	1.44	1.48	1.45	1.43	1.36	1.40	1.45
Phosphorus	0.56	0.57	0.51	0.61	0.60	0.57	0.57	0.61	0.63

^1^ Digestible energy was evaluated, and other chemical composition was measured.

### 2.2. Sample Collection

Feed samples were obtained at the start of the experiment and preserved under differentiated temperature conditions: −20 °C for subsequent chemical analysis and −80 °C for specialized fatty acid (FA) assessment. At the end of the trial, blood samples were obtained and processed into serum for subsequent FA analysis, lipid metabolism enzyme activity, antioxidant activities, immune signaling molecule levels, and microorganism analysis. Withholding food for 12 h and water for 2 h preceded slaughter. Following slaughter, samples of liver, longissimus thoracis muscle, and subcutaneous adipose tissue were collected from all donkeys and stored at −80 °C for subsequent analysis of FA content, lipid metabolism enzyme activities, and mRNA expression levels of lipid metabolism-related genes, including *PPARγ*. Liver was also used for metabolome analysis, and its homogenates were prepared for antioxidant activities and immune signaling molecule level analysis.

### 2.3. Fatty Acid Contents in Longissimus Dorsi Muscle, Subcutaneous Fat, Serum, and Liver

Fatty acid methyl esters were prepared from distinct sample matrices, including feed and longissimus dorsi muscle (0.5 g each), subcutaneous fat (0.05 g), serum (1.2 mL), and liver (0.3 g), following the protocol described by O’Fallon et al. [14], and subsequently analyzed as previously reported [15]. Thirty-seven single FAs were measured in the diet, longissimus thoracis muscle, subcutaneous adipose tissue, and liver and calculated as SFA, unsaturated fatty acids (UFAs), MUFA, PUFA, n-3 PUFA, n-6 PUFA, n6/n3, U/S, and P/S.

### 2.4. Lipid Metabolism Enzyme Content

The content of FA synthase (FAS), acetyl-CoA carboxylase (ACC), lipoprotein lipase (LPL), hormone-sensitive lipase (HSL), stearoyl-CoA desaturase (SCD), elongation of very long chain FA protein (ELOVL) 2, ELOVL5, solute carrier family 27 member 4 (SLC27A4) in serum, longissimus dorsi muscle, subcutaneous fat, and liver of meat donkeys was evaluated using commercial double-antibody one-step sandwich ELISA kits (RD Company, Minneapolis, MN, USA) following the manufacturer’s protocols.

### 2.5. Lipid Metabolism Genes and PPARγ mRNA Expression

TRIzol reagent (TaKaRa, Dalian, China) was used in accordance with the manufacturer’s instructions to extract the total RNA from the longissimus thoracis muscle, subcutaneous adipose tissue, serum, and liver specimens. Following the manufacturer’s instructions once more, the extracted RNA was reverse-transcribed into complementary DNA (cDNA) using the PrimeScript RT Reagent Kit (Yisheng Biotechnology Co., Ltd., Beijing, China). We performed quantitative real-time PCR analysis on a LightCycler 480 platform (Roche AG, Basel, Switzerland) using SYBR Green Premix Ex Taq II (Yisheng Biotechnology Co., Ltd.) to evaluate gene expression patterns in lipid metabolism, including *FAS*, *ACACA*, *LPL*, *LIPE*, *SCD*, *ELOVL2*, *ELOVL5*, *FADS1*, and *PPARγ*. Glyceraldehyde-3-phosphate dehydrogenase (*GAPDH*) and β-actin (*ACTB*) were used as internal reference genes for standardization. Table S1 lists every primer sequence that was created using the NCBI Primer-Blast online tool (https://www.ncbi.nlm.nih.gov/tools/primer-blast/ accessed on 21 October 2024). The target gene mRNA levels were quantified using the 2^−∆∆^^CT^ method, which follows the relative comparative threshold cycle technique outlined in previous literature [16]. To normalize the qRT-PCR data, we took the geometric mean of the Ct values from two reference genes [17].

### 2.6. Antioxidant Activities and Immune Signaling Molecule Levels in Serum and Liver

Using commercially available assay kits from the Nanjing Jiancheng Bioengineering Institute in Nanjing, China, we measured the concentrations of essential antioxidant enzymes and oxidative stress indicators in serum and cecum, specifically catalase (CAT, A007-1-1), glutathione peroxidase (GPx, A005-1-2), total superoxide dismutase (T-SOD, A001-1-2), and malondialdehyde (MDA, A003-1-2). Using corresponding ELISA kits provided by the Beijing Sinouk Institute of Biological Technology in Beijing, China, we simultaneously measured levels of interleukin (IL)-1β, IL-2, IL-6, IL-4, IL-10, tumor necrosis factor-alpha (TNF-α), nitric oxide (NO), and reactive oxygen species (ROS).

### 2.7. Serum and Liver Metabolome Analysis

Liquid chromatography-tandem mass spectrometry (LC-MS/MS) was used to characterize the metabolome of serum (*n* = 24) and liver (*n* = 24). Low-temperature ultrasonication was used to extract samples of liver tissue (50 mg) or serum (100 μL). The supernatant was collected for further LC-MS/MS analysis after centrifugation. An HSS T3 column (100 mm × 2.1 mm, 1.8 μm) and a Thermo Fisher Scientific UHPLC-Q Exactive system (Waltham, MA, USA) were used to perform the separation. The mobile phases consisted of 0.1% formic acid in water: acetonitrile (95:5, *v*/*v*) (solvent A) and 0.1% formic acid in acetonitrile: isopropanol: water (47.5:47.5:5, *v*/*v*) (solvent B). The flow rate was set to 0.4 mL/min. The column temperature was maintained at 40 °C. The mass spectrometric data were collected using a Thermo UHPLC-Q Exactive Mass Spectrometer (Waltham, MA, USA) equipped with an electrospray ionization (ESI) source operating in either positive or negative ion mode: −2800 V in negative mode and 3500 V in positive mode, respectively. The detection was carried out over a mass range of 70–1050 *m*/*z*. A pooled quality control (QC) sample was created by combining equal volumes of each experimental sample and processing it in the same way as the analytical samples to assess the stability of the analytical procedure. The QC sample was inserted every eight consecutive sample runs during the LC/MS analysis. Variables with relative standard deviation (RSD) > 30% of QC samples were removed, and log10 logarithmization was performed to obtain the final data matrix for subsequent analysis. The raw data were preprocessed using Progenesis QI software (V.3.0, Waters Corporation, Milford, MA, USA). Metabolites were found by searching the HMDB, Metlin, and Majorbio databases. All processed data were then uploaded to the Majorbio Cloud Platform (https://cloud.majorbio.com, accessed on 21 October 2024) for statistical analysis.

### 2.8. Statistical Analysis

The statistical significance of data was evaluated by SAS 9.0, using the ANOVA procedure on normally distributed data, otherwise using the Kruskal–Wallis test. Differences among treatment means were analyzed by the Tukey–Kramer method. Differences among the mean values were considered significant at *p* < 0.05. Differential metabolites were identified based on the variable importance in projection (VIP) values derived from the OPLS-DA model and the *p*-values from Student’s *t*-test. Metabolites with VIP > 1 and *p* < 0.05 were considered statistically significant. The magnitude of change in these metabolites was assessed using fold-change (FC) analysis, where an FC > 1 indicates upregulation, and FC < 1 indicates downregulation.

## 3. Results

### 3.1. Fatty Acid Contents

As shown in Table 2, C18:2n6t concentration in the longissimus thoracis muscle was substantially greater in the MEG than in the LEG (*p* = 0.014). There were no discernible changes between the LEG and the MEG or HEG. The HEG had a considerably larger level of C18:2n6c in the longissimus thoracis muscle, P/S ratio in subcutaneous adipose, and levels of C20:3n3 in liver than the LEG (*p* = 0.045, *p* = 0.020, *p* = 0.013), but neither of these two groups differed significantly from the MEG. The HEG had considerably greater content of C18:3n3 in the longissimus thoracis muscle, concentrations of C18:3n3, PUFA, n-3 PUFA, and n-6 PUFA in subcutaneous adipose, levels of C18:2n6t and C18:2n6c in serum, C18:2n6c, PUFA, n-6 PUFA, and U/S and P/S ratios in the liver than the LEG and MEG (*p* < 0.05). There was no discernible statistical difference between the MEG and LEG. The concentrations of C20:3n3 and n-6PUFAs in the longissimus thoracis muscle, concentration of C18:2n6t in subcutaneous adipose, contents of C18:3n3 and n-3 PUFA in serum, and content of C20:5n3 in the liver were considerably higher in the MEG and HEG as compared to the LEG (*p* < 0.05), with no meaningful difference between the MEG and HEG. C22:2n6 and the n-6/n-3 ratio in subcutaneous adipose, C18:3n6 and the n-6/n-3 ratio in serum, and the n-6/n-3 ratio in the liver were opposite. There was a substantial rise in n-3PUFA content in the longissimus thoracis muscle, C18:2n6c content in subcutaneous adipose, PUFA and n-6 PUFA contents in serum, and a significant drop in the n-6/n-3 ratio in the longissimus thoracis muscle (*p* < 0.05), which occurred alongside an increase in dietary energy intake. In comparison to the MEG, the HEG had considerably greater U/S ratios in the longissimus thoracis muscle (*p* = 0.013). Numerous non-dominant fatty acids also exhibited significant alterations in response to varying dietary energy levels; complete data for these are available in Supplementary Tables S3–S6.

**Table 2:** Effects of dietary energy level on the unsaturated fatty acid composition of the different tissues of meat donkeys (g/100 g total fatty acid).

Tissue	Fatty Acids	LEG	MEG	HEG	SEM	*p*-Value
longissimus thoracis muscle	n-6PUFA					
	C18:2n6t	0.042 ^b^	0.058 ^a^	0.051 ^ab^	0.003	0.014
	C18:2n6c	24.936 ^b^	26.322 ^ab^	27.169 ^a^	0.593	0.045
	C18:3n6	0.038	0.034	0.038	0.002	0.374
	C22:2n6	0.018	0.018	0.018	0.002	0.999
	n-3PUFA					
	C18:3n3	1.348 ^b^	1.506 ^b^	1.809 ^a^	0.074	0.001
	C20:3n3	0.130 ^b^	0.242 ^a^	0.218 ^a^	0.028	0.033
	C20:5n3	0.053	0.068	0.050	0.011	0.974
	Sum and Ratio ^1^					
	PUFA	27.700	28.300	29.104	0.484	0.145
	n-3PUFA	1.590 ^c^	1.894 ^b^	2.185 ^a^	0.076	<0.001
	n-6PUFA	25.312 ^b^	27.410 ^a^	27.394 ^a^	0.609	0.036
	n-6/n-3	15.094 ^a^	13.825 ^b^	12.063 ^c^	0.387	<0.001
	U/S	1.699 ^ab^	1.640 ^b^	1.817 ^a^	0.043	0.026
	P/S	0.732	0.752	0.834	0.035	0.124
subcutaneous adipose	n-6PUFA					
	C18:2n6t	0.008 ^b^	0.012 ^a^	0.012 ^a^	0.001	<0.001
	C18:2n6 c	25.436 ^c^	26.641 ^b^	29.392 ^a^	0.379	<0.001
	C18:3n6	0.022	0.021	0.021	0.001	0.800
	C22:2n6	0.025 ^a^	0.018 ^b^	0.020 ^b^	0.001	0.004
	n-3PUFA					
	C18:3n3	2.800 ^b^	3.140 ^b^	4.094 ^a^	0.165	<0.001
	C20:3n3	0.077	0.055	0.052	0.010	0.165
	C20:5n3	0.009	0.011	0.009	0.001	0.221
	Sum and Ratio ^1^					
	PUFA	29.421 ^b^	30.090 ^b^	34.419 ^a^	0.340	<0.001
	n-3PUFA	2.917 ^b^	3.229 ^b^	4.171 ^a^	0.167	<0.001
	n-6PUFA	26.446 ^b^	26.399 ^b^	30.335 ^a^	0.473	<0.001
	n-6/n-3	9.174 ^a^	8.166 ^b^	7.781 ^b^	0.236	0.005
	U/S	2.094	2.117	2.123	0.083	0.968
	P/S	0.910 ^b^	0.980 ^ab^	1.079 ^a^	0.039	0.020
serum	n-6PUFA					
	C18:2n6t	0.215 ^b^	0.210 ^b^	0.370 ^a^	0.028	0.002
	C18:2n6c	37.510 ^b^	38.487 ^b^	39.962 ^a^	0.351	<0.001
	C18:3n6	0.050 ^a^	0.021 ^b^	0.007 ^b^	0.006	<0.001
	C22:2n6	0.227	0.215	0.212	0.013	0.660
	n-3PUFA					
	C18:3n3	0.732 ^b^	0.983 ^a^	0.910 ^a^	0.041	0.001
	C20:3n3	0.127	0.146	0.180	0.017	0.108
	C20:5n3	0.131	0.148	0.147	0.014	0.624
	Sum and Ratio ^1^					
	PUFA	40.319 ^c^	42.078 ^b^	43.687 ^a^	0.375	<0.001
	n-3PUFA	1.122 ^b^	1.346 ^a^	1.368 ^a^	0.059	0.013
	n-6PUFA	39.197 ^c^	40.740 ^b^	42.311 ^a^	0.446	<0.001
	n-6/n-3	35.661 ^a^	32.853 ^b^	32.630 ^b^	0.638	0.005
	U/S	1.873	1.582	1.602	0.168	0.939
	P/S	1.118	1.103	1.135	0.069	0.330
liver	n-6PUFA					
	C18:2n6t	0.057	0.060	0.078	0.007	0.488
	C18:2n6c	40.943 ^b^	41.704 ^b^	43.758 ^a^	0.396	<0.001
	C18:3n6	0.028	0.030	0.032	0.002	0.400
	C22:2n6	0.035	0.028	0.036	0.003	0.099
	n-3PUFA					
	C18:3n3	0.859	1.053	1.212	0.112	0.105
	C20:3n3	0.102 ^b^	0.118 ^ab^	0.137 ^a^	0.007	0.013
	C20:5n3	0.015 ^b^	0.028 ^a^	0.033 ^a^	0.003	0.001
	Sum and Ratio ^1^					
	PUFA	44.01 ^b^	44.95 ^b^	47.54 ^a^	0.387	<0.001
	n-3PUFA	1.020	1.240	1.390	0.115	0.067
	n-6PUFA	42.99 ^b^	43.70 ^b^	46.10 ^a^	0.455	<0.001
	n-6/n-3	45.63 ^a^	35.99 ^b^	32.81 ^b^	2.126	0.001
	U/S	1.343 ^b^	1.447 ^b^	1.678 ^a^	0.055	0.001
	P/S	1.031 ^b^	1.100 ^b^	1.415 ^a^	0.076	<0.001

PUFA = polyunsaturated fatty acid; n-6/n-3 = n-6 PUFA/n-3 PUFA; U/S = unsaturated fatty acid/SFA; P/S = PUFA/SFA. LEG = low-energy group. MEG= medium-energy group. HEG = high-energy group. SEM = standard error of least squares means. ^abc^ At *p* < 0.05, means in the same row that are followed by the same superscript letters do not differ substantially.

### 3.2. Lipid Metabolism Enzyme Activity

As shown in Table 3, the longissimus dorsi muscle’s LPL activity was considerably greater in the HEG than in the LEG (*p* = 0.009). Both the MEG and HEG showed considerably higher ELOVL5 content in the longissimus dorsi muscle as compared to the LEG (*p* = 0.011). The contents of ELOVL5 in the liver and ACC and SCD in subcutaneous fat showed similar patterns. The HEG exhibited significantly higher serum content of ACC, LPL, FAS, SCD, ELOVL5, and SLC27A4 versus the LEG and MEG (*p* < 0.05). In contrast, HSL content was significantly lower in subcutaneous fat, serum, and liver tissues of the HEG (*p* = 0.002, *p* = 0.001, *p* = 0.034).

### 3.3. Lipid Metabolism Genes and PPARγ mRNA Expression

As shown in Table 4, relative to the LEG, both the MEG and HEG exhibited a significant upregulation in the expression of *PPARγ* and *ELOVL5* in the longissimus dorsi muscle and liver (*p* < 0.05). The mRNA expression of *SCD*, *FASN*, and *ACACA* in subcutaneous adipose tissue showed a similar increasing trend. *FADS1* expression in the longissimus dorsi muscle was substantially higher in the MEG than in the other two groups (*p* < 0.001). Additionally, the HEG had markedly higher levels of *PPARγ* in subcutaneous adipose tissue and *FASN* and *LIPE* in the liver versus the LEG (*p* = 0.049, *p* = 0.013, *p* = 0.002).

### 3.4. Antioxidant Activities and Immune Signaling Molecule Levels in Serum and Liver

Additionally, our findings demonstrated that dietary energy levels exerted significant impacts on antioxidant enzyme activities and immune signaling factors in both serum and liver of donkeys (Table 5 and Table 6). As shown in the tables, compared with the LEG, MEG and HEG markedly improved CAT activity and NO content in serum and liver (*p* = 0.001, *p* < 0.001) while decreasing T-SOD activity. Compared to the LEG and HEG, the MEG significantly increased serum GPx activity, hepatic GPx activity, T-SOD activity, and IL-10 levels, with respective increases of 13.45% and 11.43%, 13.24% and 11.77%, 0.69% and 7.39%, and 14.00% and 8.8% versus LEG and HEG. Concurrently, MEG reduced serum levels of IL-1β, MDA, and ROS, as well as hepatic levels of IL-1β, IL-2, and IL-6, showing respective decreases of 23.79% and 19.91%, 12.50% and 12.50%, 9.79% and 8.82%, 28.29% and 29.48%, 22.84% and 19.40%, and 14.72% and 21.76% compared to LEG and HEG. Hepatic MDA content and serum IL-2 and IL-6 levels were considerably greater in the MEG versus the LEG (*p* = 0.001, *p* < 0.001, *p* < 0.001). In comparison to the LEG, these metrics were considerably lower in the HEG. Serum IL-4 showed the opposite changing trend from these indices. Both the MEG and HEG had significantly higher serum IL-10 content than the LEG (*p* < 0.001), with the MEG showing a higher concentration (*p* < 0.001). The MEG’s hepatic TNF-α levels were substantially lower than those of the HEG (*p* = 0.019); however, they did not alter significantly between the LEG and either the MEG or HEG. Additionally, as dietary energy levels increased, serum TNF-α levels considerably increased (*p* < 0.001).

### 3.5. Serum Metabolome

The PCA demonstrated clear separation of the serum metabolome across the three groups (Figure S1A,B), with most samples located within the 95% confidence interval. A model-based analysis further confirmed statistically significant metabolic differences among the groups. For MEG vs. LEG, the positive model detected 15 differential peaks (Figure S1C), while the negative model identified 38 peaks (Figure S1D), corresponding to 53 differential metabolites (DMs) (33 downregulated, 20 upregulated). For HEG vs. LEG, the positive model screened 43 differential peaks (Figure S1E), whereas the negative model revealed a higher number of peaks (91, Figure S1F), associated with 134 DMs (83 downregulated, 41 upregulated). In MEG vs. HEG, comparative analysis showed 15 differential peaks in the positive model (Figure S1G) and 49 peaks in the negative model (Figure S1H), yielding 64 DMs (21 downregulated, 43 upregulated). The Kyoto Encyclopedia of Genes and Genomes (KEGG, http://www.genome.jp/kegg/pathway.html, accessed on 4 June 2023) database was used to assess differential metabolites found through intergroup comparisons for metabolic enrichment and pathway mapping. This approach enabled the annotation and visualization of associated biochemical pathways. Subsequent statistical evaluation was performed using Scipy.stats (a Python package; https://docs.scipy.org/doc/scipy/, accessed on 4 June 2023), and Fisher’s exact test was applied to determine the biological pathways most significantly associated with the experimental treatment. It revealed three, fifteen, and ten differentially enriched metabolic pathways in the MEG vs. LEG, HEG vs. LEG, and MEG vs. HEG, respectively (Table 7), including pentose and glucuronate interconversions (*p* = 0.109), D-Glutamine and D-glutamate metabolism (*p* = 0.001), and Aminoacyl-tRNA biosynthesis (*p* = 0.031).

### 3.6. Liver Metabolome

PCA demonstrated distinct clustering of hepatic metabolic profiles across the three experimental groups (MEG, HEG, LEG), with samples predominantly localized within the 95% confidence intervals (Figure S2A,B). Multivariate statistical modeling further confirmed pronounced intergroup metabolic heterogeneity. Specifically, MEG vs. LEG comparisons identified 31 and 30 differential peaks in positive- and negative-ion modes, respectively (Figure S2C,D), corresponding to 61 differentially regulated metabolites (DMs; 28 downregulated, 33 upregulated). Analogously, HEG vs. LEG analyses revealed a more substantial disparity, with 166 positive-mode peaks and 109 negative-mode peaks (Figure S2E,F) mapping to 285 DMs (151 downregulated, 124 upregulated). MEG vs. HEG differential profiles exhibited intermediate complexity, showing 123 positive-mode and 64 negative-mode peaks (Figure S2G,H) associated with 187 DMs (97 downregulated, 90 upregulated). Identified DMs were annotated using the KEG pathway database (v2023, accessed 4 June 2023) and subjected to pathway enrichment analysis via Scipy.stats Python module (v1.10.0). This revealed significantly altered metabolic pathways across comparisons: MEG vs. LEG (3 pathways), HEG vs. LEG (15 pathways), and MEG vs. HEG (10 pathways; Table 8). Notably perturbed pathways included glycerophospholipid metabolism (*p* < 0.001), purine metabolism (*p* = 0.002), and glutathione metabolism (*p* = 0.007), highlighting dysregulation in lipid remodeling, nucleotide homeostasis, and oxidative stress responses.

### 3.7. Correlations Between Serum or Liver Metabolites and FA, Lipid Metabolism Enzyme Activity, Lipid Metabolism Enzyme mRNA Expression, and Antioxidant Activities and Immune Signaling Molecule Levels

**Figure 1:**
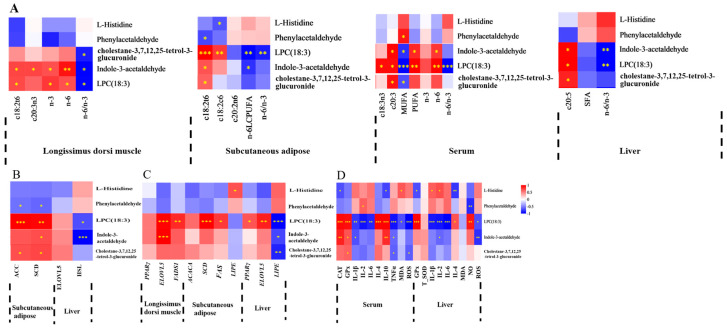
Spearman’s correlation between serum differential metabolites in MEG vs. LEG and (**A**) FA, (**B**) lipid metabolism enzyme activity, (**C**) lipid metabolism enzyme mRNA expression, (**D**) antioxidant activities, and immune signaling molecule levels. SFA = saturated fatty acid, MUFA = monounsaturated fatty acid; PUFA = polyunsaturated fatty acid; LCPUFA = long-chain polyunsaturated fatty acid; n-6/n-3 = n-6 PUFA/n-3 PUFA. FAS = fatty acid synthase, ACC = acetyl-CoA carboxylase, LPL = lipoprotein lipase, HSL = hormone-sensitive lipase, SCD = stearoyl-CoA desaturase, ELOVL = elongation of very long chain fatty acids protein, SLC27A4 = solute carrier family 27 member 4. *ACACA* = acetyl-CoA carboxylase alpha, *FADS1* = fatty acid desaturase 1, *PPARγ* = peroxisome proliferator-activated receptor γ. CAT = catalase; GPx = glutathione peroxidase; T-SOD = total superoxide dismutase; MDA = malondialdehyde; IL = interleukin; TNF-α = tumor necrosis factor-alpha; NO = nitric oxide; and ROS = reactive oxygen species. The correlation or difference between groups with significance is represented as *** *p* < 0.001, ** *p* < 0.01, * *p* < 0.05.

**Figure 2:**
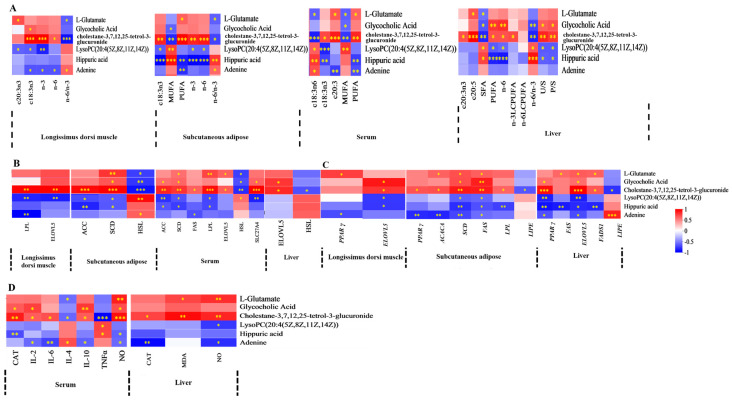
Spearman’s correlation between serum differential metabolites in HEG vs. LEG and (**A**) FA, (**B**) lipid metabolism enzyme activity, (**C**) lipid metabolism enzyme mRNA expression, (**D**) antioxidant activities, and immune signaling molecule levels. SFA = saturated fatty acid, MUFA = monounsaturated fatty acid; PUFA = polyunsaturated fatty acid; LCPUFA = long-chain polyunsaturated fatty acid; n-6/n-3 = n-6 PUFA/n-3 PUFA. FAS = fatty acid synthase, ACC = acetyl-CoA carboxylase, LPL = lipoprotein lipase, HSL = hormone-sensitive lipase, SCD = stearoyl-CoA desaturase, ELOVL = elongation of very long chain fatty acids protein, SLC27A4 = solute carrier family 27 member 4. *ACACA* = acetyl-CoA carboxylase alpha, *FADS1* = fatty acid desaturase 1, *PPARγ* = peroxisome proliferator-activated receptor γ. CAT = catalase; MDA = malondialdehyde; IL = interleukin; TNF-α = tumor necrosis factor-alpha; NO = nitric oxide. The correlation or difference between groups with significance is represented as *** *p* < 0.001, ** *p* < 0.01, * *p* < 0.05.

**Figure 3:**
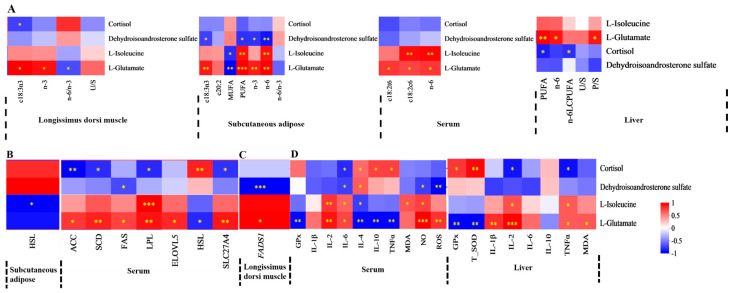
Spearman’s correlation between serum differential metabolites in MEG vs. HEG and (**A**) FA, (**B**) lipid metabolism enzyme activity, (**C**) lipid metabolism enzyme mRNA expression, (**D**) antioxidant activities, and immune signaling molecule levels. MUFA = monounsaturated fatty acid; PUFA = polyunsaturated fatty acid; LCPUFA = long-chain polyunsaturated fatty acid; n-6/n-3 = n-6 PUFA/n-3 PUFA. FAS = fatty acid synthase, ACC = acetyl-CoA carboxylase, LPL = lipoprotein lipase, HSL = hormone-sensitive lipase, SCD = stearoyl-CoA desaturase, ELOVL = elongation of very long chain fatty acids protein, SLC27A4 = solute carrier family 27 member 4. *ACACA* = acetyl-CoA carboxylase alpha, *FADS1* = fatty acid desaturase 1, *PPARγ* = peroxisome proliferator-activated receptor γ. CAT = catalase; GPx = glutathione peroxidase; T-SOD = total superoxide dismutase; MDA = malondialdehyde; IL = interleukin; TNF-α = tumor necrosis factor-alpha; NO = nitric oxide; and ROS = reactive oxygen species. The correlation or difference between groups with significance is represented as *** *p* < 0.001, ** *p* < 0.01, * *p* < 0.05.

**Figure 4:**
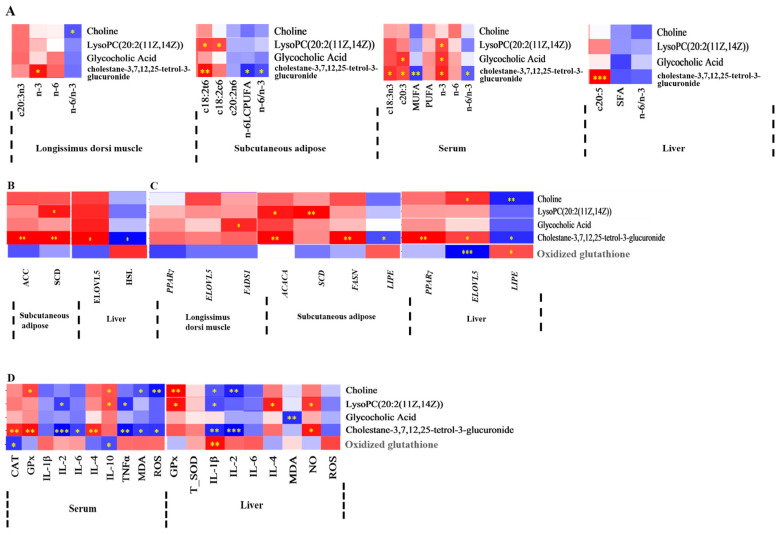
Spearman’s correlation between liver differential metabolites in MEG vs. LEG and (**A**) FA, (**B**) lipid metabolism enzyme activity, (**C**) lipid metabolism enzyme mRNA expression, (**D**) antioxidant activities, and immune signaling molecule levels. SFA = saturated fatty acid, MUF A = monounsaturated fatty acid; PUFA = polyunsaturated fatty acid; LCPUFA = long-chain polyunsaturated fatty acid; n-6/n-3 = n-6 PUFA/n-3 PUFA. FAS = fatty acid synthase, ACC = acetyl-CoA carboxylase, HSL = hormone-sensitive lipase, SCD = stearoyl-CoA desaturase, ELOVL = elongation of very long chain fatty acids protein, SLC27A4 = solute carrier family 27 member 4. ACACA = acetyl-CoA carboxylase alpha, FADS1 = fatty acid desaturase 1, PPARγ =peroxisome proliferator-activated receptor γ. CAT = catalase; GPx = glutathione peroxidase; T-SOD = total superoxide dismutase; MDA = malondialdehyde; IL = interleukin; TNF-α = tumor necrosis factor-alpha; NO = nitric oxide; and ROS = reactive oxygen species. The correlation or difference between groups with significance is represented as *** *p* < 0.001, ** *p* < 0.01, * *p* < 0.05.

**Figure 5:**
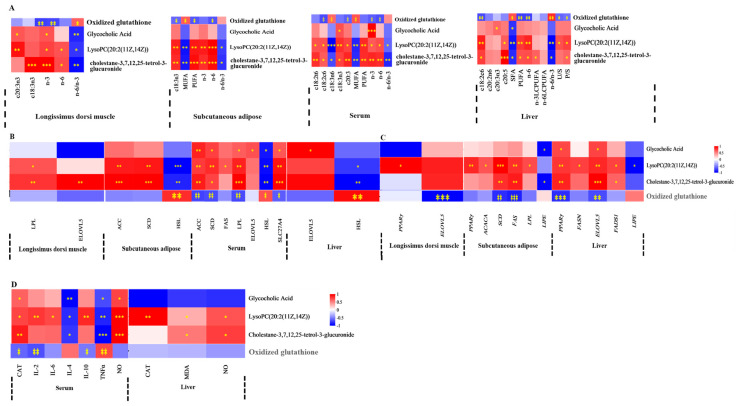
Spearman’s correlation between liver differential metabolites in HEG vs. LEG and (**A**) FA, (**B**) lipid metabolism enzyme activity, (**C**) lipid metabolism enzyme mRNA expression, (**D**) antioxidant activities, and immune signaling molecule levels. SFA = saturated fatty acid, MUFA = monounsaturated fatty acid; PUFA = polyunsaturated fatty acid; LCPUFA = long-chain polyunsaturated fatty acid; n-6/n-3 = n-6 PUFA/n-3 PUFA. FAS = fatty acid synthase, ACC = acetyl-CoA carboxylase, LPL = lipoprotein lipase, HSL = hormone-sensitive lipase, SCD = stearoyl-CoA desaturase, ELOVL = elongation of very long chain fatty acids protein, SLC27A4 = solute carrier family 27 member 4. *ACACA* = acetyl-CoA carboxylase alpha, *FADS1* = fatty acid desaturase 1, *PPARγ* = peroxisome proliferator-activated receptor γ. CAT = catalaseT-SOD = total superoxide dismutase; MDA = malondialdehyde; IL = interleukin; TNF-α = tumor necrosis factor-alpha; NO = nitric oxide. The correlation or difference between groups with significance is represented as *** *p* < 0.001, ** *p* < 0.01, * *p* < 0.05.

**Figure 6:**
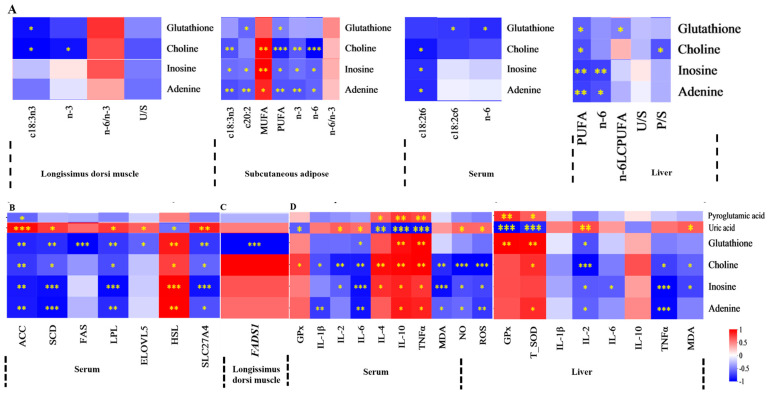
Spearman’s correlation between liver differential metabolites in MEG vs. HEG and (**A**) FA, (**B**) lipid metabolism enzyme activity, (**C**) lipid metabolism enzyme mRNA expression, (**D**) antioxidant activities, and immune signaling molecule levels. SFA = saturated fatty acid, MUFA = monounsaturated fatty acid; PUFA = polyunsaturated fatty acid; LCPUFA = long-chain polyunsaturated fatty acid; n-6/n-3 = n-6 PUFA/n-3 PUFA. FAS = fatty acid synthase, ACC = acetyl-CoA carboxylase, LPL = lipoprotein lipase, HSL = hormone-sensitive lipase, SCD = stearoyl-CoA desaturase, ELOVL = elongation of very long chain fatty acids protein, SLC27A4 = solute carrier family 27 member 4. *ACACA* = acetyl-CoA carboxylase alpha, *FADS1* = fatty acid desaturase 1, *PPARγ* = peroxisome proliferator-activated receptor γ. CAT = catalase; GPx = glutathione peroxidase; T-SOD = total superoxide dismutase; MDA = malondialdehyde; IL = interleukin; TNF-α = tumor necrosis factor-alpha; NO = nitric oxide; and ROS = reactive oxygen species. The correlation or difference between groups with significance is represented as *** *p* < 0.001, ** *p* < 0.01, * *p* < 0.05.

Spearman’s correlation coefficient was utilized to evaluate the relationships between FA composition, lipid metabolism enzyme activities, mRNA expression levels of lipid metabolism-related enzymes, antioxidant activities, immune signaling molecule concentrations, and serum or liver metabolite profiles. The findings were displayed in a correlation heatmap. The serum differential metabolite cholestane-3,7,12,25-tetrol-3-glucuronide, upregulated in MEG and HEG, demonstrated negative correlations with MUFA, c18:2n6, n-6 PUFA, and the n-6/n-3 ratio (Figure 1A and Figure 2A). Conversely, positive associations were observed with c18:3n3, c20:5n3, PUFA, and n-3 PUFA. This metabolite also showed positive correlations with both enzymatic activity and mRNA expression levels of lipid metabolism regulators (Figure 1B,C) while exhibiting favorable associations with antioxidant markers (CAT, GPx) and inverse relationships with pro-oxidative indicators (TNF-α, ROS) (Figure 2D). The oxidized glutathione in the liver was positively connected with C6: 0, n-6/n-3, IL-1β, and TNF-α but negatively with CAT and IL-10. Notably, the cortisol in serum and the oxidized glutathione in the liver showed differential regulation in MEG versus HEG, with positive associations with antioxidant enzymes and negative correlations with oxidative stress markers (Figure 3D). Hepatic differential metabolites, including choline, lysoPC(20:2(11Z,14Z)), glycocholic acid, and cholestane-3,7,12,25-tetrol-3-glucuronide, displayed consistent correlation patterns in MEG and HEG (Figure 4 and Figure 5). Liver metabolites, including pyroglutamic acid, glutathione, choline, inosine, and adenine, which were upregulated in the MEG, exhibited coordinated regulatory patterns, while uric acid was upregulated in the HEG (Figure 6D).

## 4. Discussion

### 4.1. Effects on Tissue Fatty Acid Profiles

FAs, as fundamental constituents of lipids and cellular membranes, play a vital role in determining the physicochemical properties of fats and significantly impact meat flavor profiles [18]. Key sensory characteristics of meat, such as juiciness, flavor, tenderness, and overall quality, are strongly associated with intramuscular fat (IMF) quantity and fatty acid composition [19]. A porcine study indicated that higher dietary energy intake was associated with elevated C16:1 levels and reduced concentrations of C14:0 and C17:0 in muscle tissue [20]. Dietary supplementation with high-energy rations significantly increased both total fatty acids and PUFAs in sheep meat [21]. The ratio of P/S is thought to be a key factor influencing the nutritional and health value of meat [22]. SFAs such as C12:0, C14:0, and C16:0 are known to promote cholesterol synthesis and elevate low-density lipoprotein levels, thereby increasing the risk of cardiovascular diseases [23]. Consistently, insufficient PUFA consumption is recognized as a key nutritional deficiency in suboptimal diets [24]. The n-3 series PUFAs—alpha-linolenic acid (ALA, EPA), and DHA—have been extensively investigated for decades in the context of pharma-nutrition, particularly concerning their beneficial effects on cardiovascular health [25]. Excessive intake of dietary PUFAs, especially n-6 FAs, such as linoleic acid (LA), and an unbalanced n-6/n-3 ratio contribute to metabolic disease and chronic inflammation. Dietary patterns characterized by high animal protein and n-6 PUFA intake, coupled with low n-3 PUFA consumption, have been linked to Crohn’s disease. Conversely, higher n-3 PUFA intake appears to reduce the risk of ulcerative colitis, suggesting a potentially protective role of n-3 PUFAs in inflammatory bowel disease [26]. Higher dietary energy intake improves meat quality, according to the current study. MEG and HEG improved the level of C18:3n3 in serum and C20:5n3 in the liver while reducing the n-6/n-3 ratio. What is more, compared to MEG, HEG increased the content of C18:3n3 in the longissimus dorsi muscle and subcutaneous adipose. Dietary nutrients are digested in the intestine, absorbed into the bloodstream, and subsequently transported to the liver—the central hub of FA metabolism. In this study, the FA profiles in muscle and adipose tissue exhibited consistency with those in serum and the liver, suggesting systemic metabolic coordination. Furthermore, the minor variations in dietary FA composition induced by differential energy levels may partially account for the tissue-specific differences observed in muscle and adipose tissue.

The *PPARγ* plays a central role in lipid metabolism by upregulating key lipogenic genes, including *FASN*, *ACACA*, and *LPLE*, thereby promoting lipid deposition [27]. These enzymes catalyze critical steps in de novo lipogenesis: *ACACA* initiates FA synthesis by converting acetyl-CoA to malonyl-CoA, while *FASN* drives palmitate synthesis from malonyl-CoA [28]. Notably, the liver exhibits variable responses to dietary fat due to its primary role in lipid transport and β-oxidation rather than lipogenesis [29]. Adipose tissue showed coordinated upregulation of *PPARγ*, *FASN*, and *SCD* under high-energy conditions, as observed in cattle fed high-starch diets [30] and yaks with elevated intramuscular fat deposition [6]. This metabolic adaptation is further characterized by the downregulation of *LIPE*, which limits lipolysis and enhances lipid retention. The endogenous synthesis of LCPUFAs and the unsaturation degree of biomembranes are predominantly regulated by FADS1, ELOVL2, and ELOVL5; specifically, C18:3n6 undergoes elongation via ELOVL enzymes to form C20:3n6. This intermediate is then desaturated by FADS1 through divergent pathways, yielding C20:4n6. Subsequent elongation of C20:5n3 by ELOVL5 produces C22:5n3 [31]. In the present study, higher dietary energy increased the content of ACC, SCD, LPL, FAS, and ELOVL5 and their mRNA expressions, and the mRNA expression of *PPARγ* in Longissimus dorsi muscle and Subcutaneous adipose, with this consistent metabolic pattern extending to serum and hepatic compartments. Collectively, the transcriptional synergy among *PPARγ* and its target genes (*FASN*, *ACACA*, *LPL*), coupled with suppression of *LIPE*, indicates a shift toward enhanced de novo FA synthesis and tissue-specific lipid accumulation under energy-surplus conditions. MEG and HEG modulated FA composition in body tissues by enhancing lipogenic anabolism through the upregulation of desaturase and elongase activities and their gene expression while suppressing catabolic enzyme pathways, thereby reprogramming serum and hepatic lipid profiles. Additionally, HEG increased the content of C18:3n3 in longissimus dorsi muscle and subcutaneous adipose tissue, which may be attributed to the enhanced activities of ACC, SCD, and ELOVL5 in serum, leading to elevated serum levels of PUFA. This mechanism could further promote the delivery of PUFA to muscle and adipose tissues.

### 4.2. Bile Acid Metabolism and FA Profile

Zeng et al. [32] demonstrated that rumen metabolites modulate host lipid metabolism through key pathways such as coenzyme A synthesis and NADPH-dependent FA elongation, ultimately shaping muscle FA profiles in goats. A reduction in cholestane-3,7,12,25-tetrol-3-glucuronide may suggest dysregulation in bile acid metabolism. Bile acids, crucial components of bile, are integral to lipid metabolism; their downregulation often reflects diminished lipolytic capacity. Furthermore, bile acids promote the absorption and digestion of dietary lipids [33]. The digestive tract is an integral site for FA absorption, and BA assists FA absorption by binding to FAs to form celiac particles, and BA emulsification contributes to intestinal lipid absorption [34]. Additionally, BA acts as a signaling molecule to activate specific nuclear and membrane-bound receptors, including SREBPs and PPARs, and plays a direct regulatory role in the synthesis of fats [35]. Glycocholic acid is identified as a significantly elevated intermediate in primary BA metabolism and serves as a biosynthetic precursor channeled into secondary BA metabolic pathways through network topology mapping of the bile acid regulome. In the present study, the differential metabolite cholestane-3,7,12,25-tetrol-3-glucuronide in serum and liver, and glycocholic acid in the liver, which were enriched in pentose and glucuronate interconversions, and primary bile acid biosynthesis, respectively, were upregulated in MEG vs. LEG and HEG vs. LEG. They demonstrated negative correlations with MUFA, c18:2n6, n-6 PUFA, and the n-6/n-3 ratio. Conversely, positive associations were observed with c18:3n3, c20:5n3, PUFA, and n-3 PUFA. These metabolites also showed positive correlations with both enzymatic activity (ACC, SCD, and ELOVL5 et al.) and *PPARγ* mRNA expression levels of lipid metabolism regulators. Additionally, the dual-gene editing (MSTN/FGF5) affects cellular energy balance and lipid homeostasis by upregulating the pentose and glucuronate interconversions in sheep muscle satellite cells [36]. Meanwhile, in Hu sheep fed a high-concentrate diet, primary bile acid biosynthesis facilitated dietary lipid absorption and regulated cholesterol homeostasis [37]. Collectively, these findings indicate that elevated dietary energy levels could improve FA absorption by upregulating cholestane-3,7,12,25-tetrol-3-glucuronide in pentose and glucuronate interconversions and glycocholic acid in primary BA biosynthesis, thereby indirectly increasing BA levels. This mechanism likely contributed to an optimized FA profile and a reduced n-6/n-3 PUFA ratio.

### 4.3. Impaired Energy Homeostasis and Antioxidant Deficit in LEG

In addition, in meat donkeys, low-energy diets induced a negative energy balance, which promoted the oxidation of nutrients such as fatty acids (FAs) for energy supply and subsequently triggered oxidative stress [13]. In the present study, compared to LEG, MEG increased the activities of antioxidant enzymes such as CAT and GPx in serum and liver, and decreased IL-1β, TNF-α, and ROS; HEG exhibited elevated CAT activity and decreased TNF-α levels. PUFAs suppress Th17 cell activity in ex vivo conditions and attenuate experimental colitis in vivo [38]. The carbon-carbon double bonds (C = C) present in unsaturated fatty acid (UFA) chains contribute to their antioxidant properties, thereby enhancing the health-promoting quality of meat [39]. Furthermore, this observation aligns with previous rodent studies indicating that reduced bile acid levels exacerbate oxidative stress [40]. Dietary supplementation of BA considerably enlarged the activities of SOD and GPx in both hepatic and intestinal tissues, while concurrently reducing MDA accumulation, thereby mitigating oxidative-stress-associated damage in mice [41]. In parallel, LysoPC, a bioactive metabolite generated from the partial hydrolysis of phosphatidylcholine (PC) via removal of a fatty acyl chain, is also known to suppress pro-inflammatory cell secretion [42,43]. In the present study, compared with LEG, MEG and HEG increased the PUFAs (i.e., C20:3n3 in longissimus thoracis muscle, C18:3n3 and C20:3n6 in serum, and C20:5n3 in the liver). What is more, LysoPC(20:2(11Z,14Z))-enriched glycerophospholipid metabolism, cholestane-3,7,12,25-tetrol-3-glucuronide, and glycocholic acid were upregulated in MEG vs. LEG, which were positively associated with C18:3n3, C20:5n3, PUFA, and n-3 PUFA, enzymatic activity and mRNA expression levels of lipid metabolism regulators, and antioxidant markers (CAT, GPx). Conversely, they were negatively connected with MUFA, c18:2n6, n-6 PUFA, the n-6/n-3 ratio, and pro-oxidative indicators (TNF-α, ROS). Additionally, LysoPC(20:2(11Z,14Z)) levels were noticeably elevated in the HEG relative to the LEG, demonstrating a positive correlation with CAT activity (serum and hepatic levels) and a negative connection with serum TNF-α concentrations. Additionally, oxidized glutathione, enriched in glutathione metabolism, was downregulated both in MEG vs. LEG and HEG vs. LEG. And the oxidized glutathione was positively connected with n-6/n-3, IL-1β, and TNF-α but negatively with CAT and IL-10. This observation suggests that the LEG of donkeys may have experienced negative energy balance, which was associated with increased utilization of fatty acids (FAs) for oxidative energy production and elevated ROS levels. In contrast, the MEG and HEG showed improved energy homeostasis and enhanced antioxidant capacity, as indicated by higher levels of metabolites such as cholestane-3,7,12,25-tetrol-3-glucuronide, glycocholic acid, and LysoPC(20:2(11Z,14Z)), alongside lower levels of oxidized glutathione. These observed changes are correlated with optimized FA composition and a reduced n-6/n-3 PUFA ratio, forming a set of testable associations that may help explain the underlying metabolic adaptations.

### 4.4. Metabolic Coordination and Enhanced Antioxidant Capacity in MEG

This was a hint that higher dietary energy levels can provide sufficient nutrition and energy to enhance the lipid metabolism and synthesis of FA in the liver and muscle tissues of donkeys. However, high-energy diets have been linked to systemic oxidative stress [44]. Supporting this, Hosseinian and Hasanzadeh [45] reported that high-energy diets feeding significantly elevated serum MDA, a lipid peroxidation marker, and concurrently decreased total antioxidant capacity (TAC) in domestic pigeons. Oxidative stress results from an imbalance between oxidants and antioxidants, leading to the overproduction of ROS. Excessive ROS levels can impair mitochondrial function, which in turn serves as a major cellular source of ROS, thereby creating a self-amplifying cycle that mechanistically links oxidative stress and mitochondrial dysfunction [46,47,48]. Cortisol may exhibit transient antioxidant effects by upregulating specific antioxidant enzymes (e.g., T-SOD, GPx), thereby attenuating ROS levels during acute stress exposure [49]. In our study, MEG improved the activities of CAT and GPx but decreased IL-1β, TNF-α, and ROS. Notably, the serum metabolite cortisol, enriched in glycerophospholipid metabolism, was upregulated in MEG vs. HEG, with positive associations with antioxidant enzymes and negative correlations with oxidative stress markers. Collectively, this finding suggested that the MEG diet may enhance antioxidant capacity in donkeys by upregulating cortisol levels.

Similarly, the MEG’s liver metabolites glutathione, pyroglutamic acid, and choline exhibited a coordinated regulatory pattern and were enriched in glutathione metabolism and glycerophospholipid metabolism, respectively. Low glutathione levels were associated with high oxidative stress [50]. Glutathione plays a role in mitigating postmortem oxidation of meat constituents, such as lipids and proteins, which enhances important quality attributes, including tenderness and color stability [51]. Pyroglutamic acid, 5-oxoproline, is an intermediate in GSH metabolism. Glutathione, functioning as the primary intracellular antioxidant, directly neutralizes ROS. Pyroglutamic acid indirectly potentiates cellular antioxidant capacity by preserving the glutathione pool’s stability. Studies have demonstrated that pyroglutamase deficiency leads to impaired glutathione biosynthesis, consequently triggering oxidative stress [52]. The liver is probably the primary site of choline metabolism, where it is found primarily as phosphatidylcholine [53]. Choline serves as a key methyl donor in biological systems. It undergoes oxidation to betaine, which then participates in the betaine-homocysteine S-methyltransferase (BHMT) reaction. This enzyme facilitates the methylation of homocysteine using betaine as a methyl donor, producing methionine and dimethylglycine as products. Methionine can subsequently be converted to cysteine for further metabolic utilization [54]. GSH is a tripeptide made up of glutamate, cysteine, and glycine. In the present study, glutathione enriched in glutathione metabolism and choline enriched in glycerophospholipid metabolism in MEG vs. HEG were positively related to the activities of CAT and GPx but were negatively connected with IL-1β, TNF-α, and ROS. Collectively, the coordinated regulation of glutathione, pyroglutamic acid, and choline in the MEG potentially synergistically enhances antioxidant and anti-inflammatory responses through stabilizing glutathione metabolism and supporting glycerophospholipid-mediated membrane integrity.

Numerous bioactivities, including anti-inflammatory and anti-cancer properties, have been identified for the purine nucleobase adenine. The adenosine monophosphate-activated protein kinase (AMPK) signalling pathway is the main mediator of these bioactivities [55]. Uric acid, the terminal metabolite of adenine metabolism, exhibits pro-inflammatory properties at elevated concentrations. Crystalline uric acid activates the NLRP3 inflammasome through lysosomal destabilization and potassium efflux [56], thereby triggering IL-1β-mediated pro-inflammatory cascades and exacerbating oxidative tissue damage via ROS overproduction [57]. An important modulator in the enhancement of inflammatory immunological responses is inosine, an endogenous purine metabolite synthesised by Akkermansia muciniphila and Bifidobacterium pseudolongum [58]. Qiu et al. demonstrated that the Xie Zhuo Tiao Zhi decoction effectively downregulated the expression of inflammatory markers (IL-1β, IL-6, and TNF-α), indicating alleviated inflammation, which was modulated via upregulated inosine in liver purine metabolism [59]. In our study, adenine and inosine, which were enriched in purine metabolism and upregulated in MEG, exhibited a negative relation with IL-1β, TNF-α, and ROS but a positive correlation with CAT and GPx activities. Meanwhile, uric acid, which was upregulated in HEG, was also enriched in purine metabolism. Collectively, this finding suggested that the MEG may enhance antioxidant capacity in donkeys by upregulating adenine and inosine levels and downregulating uric acid.

The FA profile of meat significantly influences its nutritional quality and relevance to human health. Current dietary recommendations therefore advocate reducing SFA intake while increasing consumption of long-chain n-3 PUFAs, based on their distinct roles in lipid metabolism and associated disease risks [60]. Donkey meat (Dezhou) in this study (MEG) exhibited significantly elevated concentrations of C18:3n3 (1.5%), total n-3 PUFAs (1.9%), and total PUFAs (28.3%) compared to Sunit lamb (0.73%, 1.49%, 10.06%) and Japanese black cattle (0.6%, 1.5%, 6.9%) [61,62]. Although the absolute content may vary due to differences in study conditions, the observed differences in proportions suggest potential advantages in the fatty acid profile of donkey meat, aligning with modern dietary recommendations.

**Figure 7:**
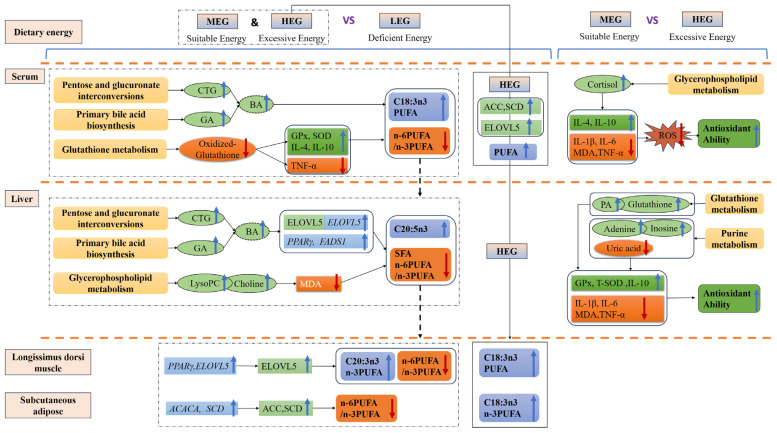
Schematic mechanism underlying dietary energy modulation of fatty acid metabolism. LEG = low-energy group. MEG = medium-energy group. HEG = high-energy group. CTG = cholestane-3,7,12,25-tetrol-3-glucuronide, GA = glycocholic acid, BA = bile acids, LysoPC = LysoPC(20:2(11Z,14Z)), ACC = acetyl-CoA carboxylase, SCD = stearoyl-CoA desaturase, ELOVL = elongation of very long chain fatty acids protein; *ACACA* = acetyl-CoA carboxylase alpha, *FADS1* = fatty acid desaturase 1, *PPARγ* = peroxisome proliferator-activated receptor γ. CAT = catalase; GPx = glutathione peroxidase; T-SOD = total superoxide dismutase; MDA = malondialdehyde; IL = interleukin; TNF-α = tumor necrosis factor-alpha; ROS = reactive oxygen species; SFA = saturated fatty acid, PUFA = polyunsaturated fatty acid. 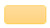 represents pathways, 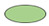 represents metabolites, 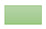 represents lipid metabolism enzyme activity, 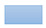 represents lipid metabolism enzyme mRNA expression, 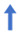 represents an increase, and 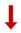 represents a decrease.

Based on these considerations, Figure 7 illustrates the mechanistic scheme of this study. The observed optimization of muscle fatty acid profiles (e.g., n-3 PUFA accumulation) in the MEG and HEG was associated with the concurrent upregulation of PPARγ and its downstream lipogenic enzyme genes (FASN/ACACA/SCD/ELOVL5). Elevated levels of bile acid metabolites (cholestane-3,7,12,25-tetrol-3-glucuronide and glycocholic acid) were also correlated with improved fatty acid profiles. It is hypothesized that these metabolites may enhance intestinal lipid emulsification or activate relevant nuclear receptor pathways, thereby potentially facilitating PUFA incorporation and reducing the n-6/n-3 ratio. Furthermore, changes in lysophosphatidylcholine (LysoPC(20:2(11Z,14Z))) and purine metabolites (adenine/hypoxanthine) showed positive correlations with increased catalase/glutathione peroxidase (CAT/GPx) activity, reduced levels of pro-inflammatory cytokines (TNF-α/IL-1β), and stabilized glutathione redox homeostasis. These associations collectively point to a potential metabolic network that may work synergistically to optimize tissue fatty acid profiles. However, although the high-energy intake (HEG) increased C18:3n3 content in muscle and adipose tissue, the elevated oxidative stress markers observed in this group suggest that this nutritional regimen may also be associated with an increased risk of oxidative vulnerability.

The present research reveals the response of donkey meat fatty acid profiles to dietary energy through integration of serum and liver metabolomics and antioxidant parameters. Only eight donkeys were selected from each group for the feeding trial due to the limitations in the selection of the test donkeys. But this trial was conducted in a single pen, where feed consumed and growth performance data were measured individually for each donkey, therefore also providing a reference value for the nutritional regulation of the fatty acid profiles of donkey meat. Subsequent studies will refine the dietary energy levels and select as many meat donkeys as possible, taking into account the breed, gender, and age, to validate the results of this trial. In this study, we only utilized Spearman correlation to correlate the measured indicators with the differential metabolites obtained from the metabolomics of serum and liver in an attempt to find a potential link between them. We have not investigated the direct effect of a specific metabolite on these indicators; some ideas and data provided reference for us to explore the related mechanisms in depth. The identification of differential metabolites and correlation analyses in this study were conducted without multiple testing correction, which increases the risk of false-positive findings. These results should therefore be interpreted as exploratory, and future validation using adjusted significance thresholds is recommended.

## 5. Conclusions

In conclusion, this study quantitatively demonstrates that dietary energy levels significantly modulate lipid metabolism, antioxidant capacity, and inflammatory tone in donkeys. Compared to the low-energy diet (LEG), both the medium-energy diet (MEG) and high-energy diet (HEG) enhanced the activity and expression of lipid-metabolizing enzymes, leading to increased levels of beneficial n-3 PUFAs (e.g., C18:3n3 in serum and fat, C20:5n3 in liver, C20:3n3 in muscle) and a reduced n-6/n-3 ratio across tissues. HEG showed a more pronounced effect on fatty acid composition, while MEG achieved a favorable balance by improving both lipid profiles and systemic antioxidant/anti-inflammatory status. Metabolomic analysis revealed that these beneficial changes were associated with the upregulation of metabolites enriched in bile acid and phospholipid metabolism pathways. However, HEG was also associated with a consistent pattern of physiological stress, indicated by decreased antioxidant metabolites (glutathione, pyroglutamic acid) and elevated pro-inflammatory markers (uric acid, TNF-α), though values remained within physiological ranges. From an applied perspective, these findings suggest a clear trade-off: while HEG more effectively optimizes fatty acid composition, it does so at the cost of heightened metabolic stress. In contrast, MEG represents a more balanced nutritional strategy that supports both meat quality and animal welfare in sustainable donkey production.

## Figures and Tables

**Table 3:** Effects of dietary energy level on contents of enzymes related to lipid metabolism in tissues and serum of meat donkey.

Items	LEG	MEG	HEG	SEM	*p*-Value
Longissimus dorsi muscle					
ACC (U/L)	27.17	28.30	28.43	0.439	0.108
LPL (U/L)	493.86 ^b^	534.35 ^ab^	563.33 ^a^	14.2	0.009
HSL (U/L)	1063.33	1021.67	1038.73	28.551	0.592
FAS (U/mL)	1581.67	1719.17	1733.25	66.687	0.230
SCD (U/L)	98.83	101.34	104.33	3.326	0.514
ELOVL2 (U/L)	81.05	81.03	81.34	1.258	0.981
ELOVL5 (U/L)	110.27 ^b^	121.43 ^a^	127.16 ^a^	3.636	0.011
SLC27A4 (U/L)	84.77	92.83	85.61	3.316	0.193
Subcutaneous adipose					
ACC (U/L)	27.71 ^b^	32.37 ^a^	32.24 ^a^	0.586	<0.001
LPL (U/L)	550.65	568.80	585.43	9.563	0.056
HSL (U/L)	1099.44 ^a^	1074.38 ^a^	958.11 ^b^	25.644	0.002
FAS (U/mL)	1696.25	1897.64	1901.56	65.508	0.061
SCD (U/L)	90.22 ^b^	97.74 ^a^	97.39 ^a^	0.778	<0.001
ELOVL2 (U/L)	72.19	72.34	72.50	1.35	0.987
ELOVL5 (U/L)	62.16	61.73	61.36	0.343	0.102
SLC27A4 (U/L)	83.64	78.23	80.66	2.173	0.234
Serum					
ACC (U/L)	38.07 ^b^	39.88 ^b^	50.82 ^a^	1.823	0.002
LPL (U/L)	470.22 ^b^	510.22 ^b^	603.26 ^a^	16.256	<0.001
HSL (U/L)	1427.22 ^a^	1385.57 ^a^	1259.86 ^b^	27.914	0.001
FAS (U/mL)	1852.78 ^b^	1926.11 ^b^	2161.06 ^a^	59.193	0.004
SCD (U/L)	132.28 ^b^	138.62 ^b^	176.98 ^a^	5.022	<0.001
ELOVL2 (U/L)	94.05	96.54	100.55	3.673	0.464
ELOVL5 (U/L)	65.15 ^b^	67.85 ^b^	78.48 ^a^	1.888	0.005
SLC27A4 (U/L)	52.23 ^b^	54.39 ^b^	68.96 ^a^	1.518	<0.001
Liver					
ACC (U/L)	31.63	31.99	33.27	0.673	0.218
LPL (U/L)	558.80	616.96	585.16	19.195	0.125
HSL (U/L)	931.96 ^a^	840.60 ^b^	849.520 ^b^	25.223	0.034
FAS (U/mL)	1856.70	1896.90	2102.70	78.669	0.083
SCD (U/L)	101.72	104.06	106.47	3.578	0.649
ELOVL2 (U/L)	89.30	91.00	89.80	1.865	0.805
ELOVL5 (U/L)	50.63 ^b^	59.87 ^a^	65.46 ^a^	2.081	<0.001
SLC27A4 (U/L)	83.95	85.36	88.04	1.786	0.280

LEG = low-energy group. MEG = medium-energy group. HEG = high-energy group. SEM = standard error of least squares means. ^ab^ At *p* < 0.05, means in the same row that are followed by the same superscript letters do not differ substantially. FAS = fatty acid synthase, ACC = acetyl-CoA carboxylase, LPL = lipoprotein lipase, HSL = hormone-sensitive lipase, SCD = stearoyl-CoA desaturase, ELOVL = elongation of very long chain fatty acids protein, SLC27A4 = solute carrier family 27 member 4.

**Table 4:** Effects of dietary energy level on gene expression of lipid metabolism in tissues and serum of meat donkey.

Items	LEG	MEG	HEG	SEM	*p*-Value
Longissimus dorsi muscle					
*PPARγ*	1.00 ^b^	1.16 ^a^	1.23 ^a^	0.043	0.004
*ACACA*	1.00	1.01	1.00	0.016	0.183
*LPL*	1.00	1.25	1.36	0.115	0.103
*LIPE*	1.00	0.92	0.92	0.07	0.297
*FAS*	1.00	1.14	1.04	0.076	0.418
*SCD*	1.00	1.06	1.21	0.079	0.172
*ELOVL2*	1.00	1.02	1.18	0.063	0.111
*ELOVL5*	1.00 ^b^	1.14 ^a^	1.14 ^a^	0.037	0.007
*FADS1*	1.00 ^b^	1.30 ^a^	0.86 ^b^	0.059	<0.001
Subcutaneous adipose					
*PPARγ*	1.00 ^b^	1.14 ^ab^	1.33 ^a^	0.077	0.049
*ACACA*	1.00 ^b^	1.28 ^a^	1.27 ^a^	0.066	0.011
*LPL*	1.00 ^b^	1.24 ^b^	1.55 ^a^	0.086	0.005
*LIPE*	1.00 ^a^	0.97 ^b^	0.85 ^b^	0.038	0.028
*FAS*	1.00 ^b^	1.26 ^a^	1.31 ^a^	0.052	0.002
*SCD*	1.00 ^b^	1.62 ^a^	1.60 ^a^	0.174	0.002
*ELOVL2*	1.00	1.12	1.17	0.067	0.199
*ELOVL5*	1.00	0.97	0.99	0.073	0.569
*FADS1*	1.00	1.08	1.03	0.059	0.605
Liver					
*PPARγ*	1.00 ^b^	1.26 ^a^	1.33 ^a^	0.082	0.002
*ACACA*	1.00	1.02	1.08	0.116	0.558
*LPL*	1.00	1.19	1.06	0.149	0.174
*LIPE*	1.00 ^a^	0.68 ^b^	0.83 ^b^	0.056	0.002
*FAS*	1.00 ^b^	1.09 ^ab^	1.16 ^a^	0.042	0.013
*SCD*	1.00	1.03	1.13	0.064	0.221
*ELOVL2*	1.00	1.13	1.04	0.069	0.553
*ELOVL5*	1.00 ^b^	1.09 ^a^	1.12 ^a^	0.025	0.002
*FADS1*	1.00 ^b^	1.09 ^ab^	1.25 ^a^	0.051	0.017

LEG = low-energy group. MEG = medium-energy group. HEG = high-energy group. SEM = standard error of least squares means. ^ab^ At *p* < 0.05, means in the same row that are followed by the same superscript letters do not differ substantially. *FAS* = fatty acid synthase, *ACACA* = acetyl-CoA carboxylase alpha, *LPL* = lipoprotein lipase, *LIPE* = hormone-sensitive lipase, *SCD* = stearoyl-CoA desaturase, *ELOVL* = elongation of very long chain fatty acids protein, *FADS1* = fatty acid desaturase 1, *PPARγ* = peroxisome proliferator-activated receptor γ.

**Table 5:** Serum antioxidant activities and immune signaling molecule levels in response to dietary energy intake.

Item	LEG	MEG	HEG	SEM	*p* Value
Antioxidant enzyme activities, U/mL					
CAT	8.26 ^b^	11.89 ^a^	11.61 ^a^	0.341	0.001
GPx	433.33 ^b^	500.67 ^a^	443.43 ^b^	13.342	0.004
T-SOD	136.76 ^a^	122.51 ^b^	119.13 ^b^	2.148	0.001
Immune signaling molecule, pg/mL					
IL-1β	26.17 ^a^	21.14 ^b^	25.35 ^a^	0.789	<0.001
IL-2	268.73 ^b^	233.55 ^c^	290.02 ^a^	4.675	<0.001
IL-6	155.97 ^b^	132.95 ^c^	168.65 ^a^	2.228	<0.001
IL-4	6.53 ^b^	7.80 ^a^	5.76 ^c^	0.147	<0.001
IL-10	8.30 ^c^	12.13 ^a^	9.42 ^b^	0.230	<0.001
TNF-α	70.48 ^a^	46.67 ^b^	35.57 ^c^	1.767	<0.001
MDA concentration, nmol/mL	2.16 ^a^	1.92 ^b^	2.16 ^a^	0.045	0.003
NO concentration, μmol/L	40.20 ^b^	39.65 ^b^	53.46 ^a^	1.601	<0.001
ROS (IU/mL)	131.00 ^a^	119.32 ^b^	129.84 ^a^	1.104	<0.001

LEG = low-energy group. MEG = medium-energy group. HEG = high-energy group. SEM = standard error of least squares means. ^abc^ At *p* < 0.05, means in the same row that are followed by the same superscript letters do not differ substantially. CAT = catalase; GPx = glutathione peroxidase; T-SOD = total superoxide dismutase; MDA = malondialdehyde; IL = interleukin; TNF-α = tumor necrosis factor-alpha; NO = nitric oxide; and ROS = reactive oxygen species.

**Table 6:** Hepatic antioxidant activities and immune signaling molecule levels in response to dietary energy intake.

Item	LEG	MEG	HEG	SEM	*p* Value
Antioxidant enzyme activities, U/mgprot.					
CAT	53.66 ^b^	57.93 ^ab^	68.29 ^a^	3.609	0.026
GPx	82.60 ^b^	95.21 ^a^	84.00 ^b^	2.527	0.004
T-SOD	89.52 ^b^	90.14 ^a^	83.48 ^b^	1.721	0.023
Immune signaling molecule, pg/mgprot.					
IL-1β	3.22 ^a^	2.51 ^b^	3.25 ^a^	0.188	0.009
IL-2	21.46 ^a^	17.47 ^b^	20.86 ^a^	0.252	<0.001
IL-6	14.34 ^a^	12.50 ^b^	15.22 ^a^	0.365	0.002
IL-4	0.78	0.95	0.85	0.046	0.058
IL-10	2.15 ^b^	2.50 ^a^	2.28 ^b^	0.073	0.010
TNF-α	2.04 ^ab^	1.89 ^b^	2.27 ^a^	0.093	0.019
MDA concentration, nmol/mgprot.	1.86 ^b^	1.59 ^c^	2.13 ^a^	0.080	0.001
NO concentration, μmol/gprot.	6.64 ^b^	7.60 ^a^	7.84 ^a^	0.178	<0.001
ROS (IU/mgprot.)	281.35	252.61	274.77	9.075	0.087

LEG = low-energy group. MEG = medium-energy group. HEG = high-energy group. SEM = standard error of least squares means. ^abc^ At *p* < 0.05, means in the same row that are followed by the same superscript letters do not differ substantially. CAT = catalase; GPx = glutathione peroxidase; T-SOD = total superoxide dismutase; MDA = malondialdehyde; IL = interleukin; TNF-α = tumor necrosis factor-alpha; NO = nitric oxide; and ROS = reactive oxygen species.

**Table 7:** KEGG pathway enrichment and metabolites annotated to the pathway in serum metabolism (impact > 0).

Metabolic Pathways	*p*-Value	Up-Metabolites	Down-Metabolites
MEG vs. LEG			
Pentose and glucuronate interconversions	0.109	Cholestane-3,7,12,25-tetrol-3-glucuronide	
HEG vs. LEG			
D-Glutamine and D-glutamate metabolism	0.001	L-Glutamate	
Glycerophospholipid metabolism	0.015	LPC(18:3)	LysoPC(20:4(5Z,8Z,11Z,14Z)) PC(18:2(9Z,12Z)/20:4(5Z,8Z,11Z,14Z))
Phenylalanine metabolism	0.017		Hippuric acid Benzoic acid
Purine metabolism	0.039	Hypoxanthine	Adenine
Taurine and hypotaurine metabolism	0.077	L-Glutamate	
Arginine biosynthesis	0.087	L-Glutamate	
Alanine, aspartate, and glutamate metabolism	0.104	L-Glutamate	
Glutathione metabolism	0.136	L-Glutamate	
Primary bile acid biosynthesis	0.159	Glycocholic Acid	
Aminoacyl-tRNA biosynthesis	0.175	L-Glutamate	
Glyoxylate and dicarboxylate metabolism	0.178	L-Glutamate	
Pentose and glucuronate interconversions	0.178	Cholestane-3,7,12,25-tetrol-3-glucuronide	
Tryptophan metabolism	0.181	Indole-3-acetaldehyde	
Arginine and proline metabolism	0.225	L-Glutamate	
MEG vs. HEG			
D-Glutamine and D-glutamate metabolism	0.002		L-Glutamate
Aminoacyl-tRNA biosynthesis	0.031		L-Isoleucine L-Glutamate
Glycerophospholipid metabolism	0.100	PC(18:2(9Z,12Z)/20:4(5Z,8Z,11Z,14Z))	
Steroid hormone biosynthesis	0.016	Cortisol Dehydroisoandrosterone sulfate	

LEG = low-energy group. MEG = medium-energy group. HEG = high-energy group.

**Table 8:** KEGG pathway enrichment and metabolites annotated to the pathway in liver metabolism (impact > 0).

Metabolic Pathways	*p*-Value	Up-Metabolites	Down-Metabolites
MEG vs. LEG			
Glycerophospholipid metabolism	0.000	Choline, LysoPC(20:2(11Z,14Z))	PS(18:0/20:4(8Z,11Z,14Z,17Z)) PS(18:0/22:5(7Z,10Z,13Z,16Z,19Z)) PC(15:0/18:2(9Z,12Z))
Glutathione metabolism	0.102		Oxidized glutathione
Primary bile acid biosynthesis	0.121	Glycocholic Acid	
Pentose and glucuronate interconversions	0.137	Cholestane-3,7,12,25-tetrol-3-glucuronide 6-Hydroxy-5-methoxyindole glucuronide	Octanoylglucuronide
Purine metabolism	0.193		Inosine
HEG vs. LEG			
Purine metabolism	0.000	Xanthine, Uric acid	Guanosine, Inosine, Adenine, ADP
Glutathione metabolism	0.001		Glutathione, Gamma-Glu-Cys Pyroglutamic acid Oxidized glutathione
Glycerophospholipid metabolism	0.003	LysoPC(20:0), LysoPC(18:0) PC(22:5(4Z,7Z,10Z,13Z,16Z)/P-18:0) LPC(18:1), LysoPC(20:2(11Z,14Z)) LysoPC(P-18:0), LysoPC(20:1(11Z)) LPC(18:3)	Dimethylethanolamine PS(18:0/20:4(8Z,11Z,14Z,17Z)) PS(18:0/22:5(7Z,10Z,13Z,16Z,19Z)) PC(16:0/16:0), PC(15:0/18:2(9Z,12Z))
Tyrosine metabolism	0.138	Phenol, L-Tyrosine	
Primary bile acid biosynthesis	0.332	Glycocholic Acid	
Pentose and glucuronate interconversions	0.350	Lithocholate 3-O-glucuronide Cholestane-3,7,12,25-tetrol-3-glucuronide	Octanoylglucuronide
MEG vs. HEG			
Purine metabolism	0.000	Guanosine, Inosine Adenosine diphosphate ribose Adenylosuccinate, Adenine, ADP	Xanthine Uric acid
Glutathione metabolism	0.007	Glutathione, Gamma-Glu-Cys Pyroglutamic acid	
Glycerophospholipid metabolism	0.014	Dimethylethanolamine, Choline	LysoPC(20:0), LPC(18:1) LysoPC(P-18:0), LPC(18:3)

LEG = low-energy group. MEG = medium-energy group. HEG = high-energy group.

## Data Availability

The readers can contact the corresponding authors as needed to request raw data. The raw sequencing data used and described in this study have been deposited into the CNGB Sequence Archive (CNSA) (https://db.cngb.org/cnsa/, accessed on 14 January 2025) of China National GeneBank Database (CNGBdb) with accession numbers CNP0007956 (Serum metabolome data, link: https://db.cngb.org/search/project/CNP0007956/, accessed on 14 January 2025) and CNP0007959 (Liver metabolome data, link: https://db.cngb.org/search/project/CNP0007959/, accessed on 14 January 2025). All information is included in the manuscript or Supplementary Materials.

## Supplementary Materials

The following supporting information can be downloaded at https://www.mdpi.com/article/10.3390/antiox15010140/s1, Table S1. Dietary composition in fattening period (air-dry basis, %). Table S2. Fatty acid composition of the experimental diet (percentage of total fatty acid). Table S3. Effects of dietary energy level on the fatty acid composition of longissimus thoracis muscle of meat donkeys (g/100g total fatty acid). Table S4. Effects of dietary energy level on the fatty acid composition of subcutaneous adipose tissue of meat donkeys (g/100g total fatty acid). Table S5. Effects of dietary energy level on serum fatty acid composition of meat donkeys (g/100g total fatty acid). Table S6. Effects of dietary energy level on fatty acid composition in liver of meat donkeys (g/100g total fatty acid). Figure S1. Serum metabolome profiles between MEG vs. LEG, HEG vs. LEG, MEG vs. HEG. Figure S2. Liver metabolome profiles between MEG vs. LEG, HEG vs. LEG, MEG vs. HEG. Table S7. Primer pairs sequences for quantitative real-time PCR.

## Abbreviations

The following abbreviations are used in this manuscript:

LEG	low-energy group
MEG	medium-energy group
HEG	high-energy group
SFA	saturated fatty acid
MUFA	monounsaturated fatty acid
PUFA	polyunsaturated fatty acid
LCPUFA	long-chain polyunsaturated fatty acid
FAS	fatty acid synthase
ACC	acetyl-CoA carboxylase
LPL	lipoprotein lipase
HSL	hormone-sensitive lipase
SCD	stearoyl-coa desaturase
ELOVL	elongation of very long chain fatty acids protein
SLC27A4	solute carrier family 27 member 4
*PPARγ*	peroxisome proliferator-activated receptor γ
*ACACA*	acetyl-coa carboxylase alpha
*LIPE*	hormone-sensitive lipase
*FADS1*	fatty acid desaturase 1
CAT	catalase
GPx	glutathione peroxidase
T-SOD	total superoxide dismutase
MDA	malondialdehyde
IL	interleukin
TNF-α	tumor necrosis factor-alpha
NO	nitric oxide
ROS	reactive oxygen species
PCA	principal component analysis

## References

[1] Polidori P., Cavallucci C., Beghelli D., Vincenzetti S. (2009). Physical and chemical characteristics of donkey meat from Martina Franca breed. Meat Sci..

[2] Wood J.D., Enser M., Fisher A.V., Nute G.R., Sheard P.R., Richardson R.I., Hughes S.I., Whittington F.M. (2008). Fat deposition, fatty acid composition and meat quality: A review. Meat Sci..

[3] Mozaffarian D., Wu J.H. (2012). (n-3) fatty acids and cardiovascular health: Are effects of EPA and DHA shared or complementary?. J. Nutr..

[4] Schumann J., Leichtle A., Thiery J., Fuhrmann H. (2011). Fatty acid and peptide profiles in plasma membrane and membrane rafts of PUFA supplemented RAW264.7 macrophages. PLoS ONE.

[5] Orr S.K., Trépanier M.O., Bazinet R.P. (2013). n-3 Polyunsaturated fatty acids in animal models with neuroinflammation. Prostaglandins Leukot. Essent. Fat. Acids.

[6] Kang K., Ma J., Wang H., Wang Z., Peng Q., Hu R., Zou H., Bao S., Zhang W., Sun B. (2020). High-energy diet improves growth performance, meat quality and gene expression related to intramuscular fat deposition in finishing yaks raised by barn feeding. Vet. Med. Sci..

[7] Yang C., Liu J., Wu X., Bao P., Long R., Guo X., Ding X., Yan P. (2017). The response of gene expression associated with lipid metabolism, fat deposition and fatty acid profile in the longissimus dorsi muscle of Gannan yaks to different energy levels of diets. PLoS ONE.

[8] Fang L.H., Jin Y.H., Do S.H., Hong J.S., Kim B.O., Han T.H., Kim Y.Y. (2019). Effects of dietary energy and crude protein levels on growth performance, blood profiles, and carcass traits in growing-finishing pigs. J. Anim. Sci. Technol..

[9] Mattijssen F., Georgiadi A., Andasarie T., Szalowska E., Zota A., Krones-Herzig A., Heier C., Ratman D., De Bosscher K., Qi L. (2014). Hypoxia-inducible lipid droplet-associated (HILPDA) is a novel peroxisome proliferator-activated receptor (PPAR) target involved in hepatic triglyceride secretion. J. Biol. Chem..

[10] Ma H., Yuan J., Ma J., Ding J., Lin W., Wang X., Zhang M., Sun Y., Wu R., Liu C. (2019). BMP7 improves insulin signal transduction in the liver via inhibition of mitogen-activated protein kinases. J. Endocrinol..

[11] Zhou Y., Zhang J., Chi Y., Yue Y.X., Zhao Y.L., Guo X.Y., Zhang Y.W., Shi B.L., Yan S.M. (2021). Effects of dietary energy level on growth, fattening performance and slaughter performance of Broiler Donkeys. Chin. J. Anim. Nutr..

[12] Du X., Zhou Y., Zhao Y.L., Guo X.Y., Guo Y.M., Zhang Y.W., Yan S.M. (2022). Effects of dietary energy level on physicochemical properties and conventional nutrient content of Donkey meat. Feed. Ind..

[13] Li L., Guo X., Zhao Y., Guo Y., Shi B., Zhou Y., Zhang Y., Yan S. (2024). Cecal Microbial Diversity and Metabolome Reveal a Reduction in Growth Due to Oxidative Stress Caused by a Low-Energy Diet in Donkeys. Antioxidants.

[14] O’Fallon J.V., Busboom J.R., Nelson M.L., Gaskins C.T. (2007). A direct method for fatty acid methyl ester synthesis: Application to wet meat tissues, oils, and feedstuffs. J. Anim. Sci..

[15] Wang X., Martin G.B., Liu S.L., Shi B.L., Guo X.Y., Zhao Y.L., Yan S.M. (2019). The mechanism through which dietary supplementation with heated linseed grain increases n-3 long-chain polyunsaturated fatty acid concentration in subcutaneous adipose tissue of cashmere kids. J. Anim. Sci..

[16] Livak K.J., Schmittgen T.D. (2001). Analysis of relative gene expression data using real-time quantitative PCR and the 2(-Delta Delta C(T)) method. Methods.

[17] Vandesompele J., De Preter K., Pattyn F., Poppe B., Van Roy N., De Paepe A., Speleman F. (2002). Accurate normalization of real-time quantitative RT-PCR data by geometric averaging of multiple internal control genes. Genome Biol..

[18] Zhan H., Cui H., Yu J., Hayat K., Wu X., Zhang X., Ho C.T. (2022). Characteristic flavor formation of thermally processed N-(1-deoxy-α-d-ribulos-1-yl)-glycine: Decisive role of additional amino acids and promotional effect of glyoxal. Food Chem..

[19] Anton I., Húth B., Füller I., Rózsa L., Holló G., Zsolnai A. (2018). Effect of single nucleotide polymorphisms on intramuscular fat content in Hungarian Simmental cattle. Asian-Australas. J. Anim. Sci..

[20] Yang C., Wang W., Tang X., Huang R., Li F., Su W., Yin Y., Wen C., Liu J. (2022). Comparison of the meat quality and fatty acid profile of muscles in finishing Xiangcun Black pigs fed varied dietary energy levels. Anim. Nutr..

[21] Vasconcelos-Filho P.T., Costa H.H.A., Vega W.H.O., Sousa L.C.O., Parente M.O.M., Landim A.V. (2021). Effects of dietary energy content and source using by-products on carcass and meat quality traits of cull ewes. Animal.

[22] Cabrera M.C., Saadoun A. (2014). An overview of the nutritional value of beef and lamb meat from South America. Meat Sci..

[23] Moloney A.P., Mooney M.T., Kerry J.P., Troy D.J. (2001). Producing tender and flavoursome beef with enhanced nutritional characteristics. Proc. Nutr. Soc..

[24] Zhou M., Wang H., Zeng X., Yin P., Zhu J., Chen W., Li X., Wang L., Wang L., Liu Y. (2019). Mortality, morbidity, and risk factors in China and its provinces, 1990–2017: A systematic analysis for the Global Burden of Disease Study 2017. Lancet.

[25] Visioli F., Poli A. (2020). Fatty Acids and Cardiovascular Risk. Evidence, Lack of Evidence, and Diligence. Nutrients.

[26] Ananthakrishnan A.N., Khalili H., Konijeti G.G., Higuchi L.M., de Silva P., Fuchs C.S., Willett W.C., Richter J.M., Chan A.T. (2014). Long-term intake of dietary fat and risk of ulcerative colitis and Crohn’s disease. Gut.

[27] Corazzin M., Bovolenta S., Saccà E., Bianchi G., Piasentier E. (2013). Effect of linseed addition on the expression of some lipid metabolism genes in the adipose tissue of young Italian Simmental and Holstein bulls. J. Anim Sci..

[28] Brownsey R.W., Boone A.N., Elliott J.E., Kulpa J.E., Lee W.M. (2006). Regulation of acetyl-CoA carboxylase. Biochem. Soc. Trans..

[29] Becker S.L., Humphrey D.C., Karriker L.A., Brown J.T., Skoland K.J., Greiner L.L. (2023). The effects of dietary essential fatty acid ratios and energy level on growth performance, lipid metabolism, and inflammation in grow-finish pigs. J. Anim. Sci..

[30] Graugnard D.E., Piantoni P., Bionaz M., Berger L.L., Faulkner D.B., Loor J.J. (2009). Adipogenic and energy metabolism gene networks in longissimus lumborum during rapid post-weaning growth in Angus and Angus x Simmental cattle fed high-starch or low-starch diets. BMC Genom..

[31] Zhang J.Y., Kothapalli K.S., Brenna J.T. (2016). Desaturase and elongase-limiting endogenous long-chain polyunsaturated fatty acid biosynthesis. Curr. Opin. Clin. Nutr. Metab. Care.

[32] Zeng Y., Mou H., He Y., Zhang D., Pan X., Zhou L., Shen Y., E G. (2024). Effects of key rumen bacteria and microbial metabolites on fatty acid deposition in goat muscle. Animals.

[33] Zhong W., Wang H., Yang Y., Zhang Y., Lai H., Cheng Y., Yu H., Feng N., Huang R., Liu S. (2022). High-protein diet prevents fat mass increase after dieting by counteracting Lactobacillus-enhanced lipid absorption. Nat. Metab..

[34] Larabi A.B., Masson H.L.P., Bäumler A.J. (2023). Bile acids as modulators of gut microbiota composition and function. Gut Microbes.

[35] Yao D., Luo J., He Q., Shi H., Li J., Wang H., Xu H., Chen Z., Yi Y., Loor J.J. (2017). SCD1 alters long-chain fatty acid (LCFA) Composition and its expression is directly regulated by SREBP-1 and PPARγ 1 in dairy goat mammary cells. J. Cell. Physiol..

[36] Chen M., Li Y., Xu X., Wang S., Liu Z., Qi S., Si D., Man Z., Deng S., Liu G. (2024). Metabolic differences in MSTN and FGF5 dual-gene edited sheep muscle cells during myogenesis. BMC Genom..

[37] Zheng K., Guo L., Cao Y., Yin Y., Gao H., Zhang X., Jiang J., Li J., Huang X., Li K. (2024). High-concentrate diet decreases lamb fatty acid contents by regulating bile acid composition. Food Chem. X.

[38] Monk J.M., Hou T.Y., Turk H.F., McMurray D.N., Chapkin R.S. (2013). n3 PUFAs reduce mouse CD4^+^ T-cell ex vivo polarization into Th17 cells. J. Nutr..

[39] Kim J., Lee E.J., Lee K.E., Nho Y.H., Ryu J., Kim S.Y., Yoo J.K., Kang S., Seo S.W. (2023). Lipid extract derived from newly isolated Rhodotorula toruloides LAB-07 for cosmetic applications. Comput. Struct. Biotechnol. J..

[40] Miller D.B., Karoly E.D., Jones J.C., Ward W.O., Vallanat B.D., Andrews D.L., Schladweiler M.C., Snow S.J., Bass V.L., Richards J.E. (2015). Inhaled ozone (O_3_)-induces changes in serum metabolomic and liver transcriptomic profiles in rats. Toxicol. Appl. Pharmacol..

[41] Guo C., Xie S., Chi Z., Zhang J., Liu Y., Zhang L., Zheng M., Zhang X., Xia D., Ke Y. (2016). Bile Acids Control Inflammation and Metabolic Disorder through Inhibition of NLRP3 Inflammasome. Immunity.

[42] Sun Y., Wang F., Liu Y., Liu S., An Y., Xue H., Wang J., Xia F., Chen X., Cao Y. (2022). Microbiome-metabolome responses of Fuzhuan brick tea crude polysaccharides with immune-protective benefit in cyclophosphamide-induced immunosuppressive mice. Food Res. Int..

[43] Li L., Fu W.W., Wu R.T., Song Y.H., Wu W.Y., Yin S.H., Li W.J., Xie M.Y. (2020). Protective effect of Ganoderma atrum polysaccharides in acute lung injury rats and its metabolomics. Int. J. Biol. Macromol..

[44] Adebowale T., Shunshun J., Yao K. (2019). The effect of dietary high energy density and carbohydrate energy ratio on digestive enzymes activity, nutrient digestibility, amino acid utilization and intestinal morphology of weaned piglets. J. Anim. Physiol. Anim. Nutr..

[45] Hosseinian S.A., Hasanzadeh F. (2021). Impact of high dietary energy on obesity and oxidative stress in domestic pigeons. Vet. Med. Sci..

[46] Chang W.L., Ko C.H. (2023). The role of oxidative stress in vitiligo: An update on its pathogenesis and therapeutic implications. Cells.

[47] Brookes P.S., Yoon Y., Robotham J.L., Anders M.W., Sheu S.S. (2004). Calcium, ATP, and ROS: A mitochondrial love-hate triangle. Am. J. Physiol. Cell Physiol..

[48] Sinha K., Das J., Pal P.B., Sil P.C. (2013). Oxidative stress: The mitochondria-dependent and mitochondria-independent pathways of apoptosis. Arch. Toxicol..

[49] Stark J., Tulassay Z., Lengyel G., Szombath D., Székács B., Adler I., Marczell I., Nagy-Répas P., Dinya E., Rácz K. (2013). Increased total scavenger capacity in rats fed corticosterone and cortisol on lipid-rich diet. Acta Physiol. Hung..

[50] Geenen S., Yates J.W., Kenna J.G., Bois F.Y., Wilson I.D., Westerhoff H.V. (2013). Multiscale modelling approach combining a kinetic model of glutathione metabolism with PBPK models of paracetamol and the potential glutathione-depletion biomarkers ophthalmic acid and 5-oxoproline in humans and rats. Integr. Biol..

[51] Mercier Y., Gatellier P., Renerre M. (2004). Lipid and protein oxidation in vitro, and antioxidant potential in meat from Charolais cows finished on pasture or mixed diet. Meat Sci..

[52] Calpena E., Casado M., Martínez-Rubio D., Nascimento A., Colomer J., Gargallo E., García-Cazorla A., Palau F., Artuch R., Espinós C. (2013). 5-Oxoprolinuria in Heterozygous Patients for 5-Oxoprolinase (OPLAH) Missense Changes. JIMD Rep..

[53] Li Z., Vance D.E. (2008). Phosphatidylcholine and choline homeostasis. J. Lipid Res..

[54] Breksa A.P., Garrow T.A. (1999). Recombinant human liver betaine-homocysteine S-methyltransferase: Identification of three cysteine residues critical for zinc binding. Biochemistry.

[55] Chen S.Y., Lin C.H., Lin J.T., Cheng Y.F., Chen H.M., Kao S.H. (2017). Adenine causes cell cycle arrest and autophagy of chronic myelogenous leukemia K562 cells via AMP-activated protein kinase signaling. Oncol. Lett..

[56] Martinon F., Pétrilli V., Mayor A., Tardivel A., Tschopp J. (2006). Gout-associated uric acid crystals activate the NALP3 inflammasome. Nature.

[57] Braga T.T., Forni M.F., Correa-Costa M., Ramos R.N., Barbuto J.A., Branco P., Castoldi A., Hiyane M.I., Davanso M.R., Latz E. (2017). Soluble Uric Acid Activates the NLRP3 Inflammasome. Sci. Rep..

[58] Mager L.F., Burkhard R., Pett N., Cooke N.C.A., Brown K., Ramay H., Paik S., Stagg J., Groves R.A., Gallo M. (2020). Microbiome-derived inosine modulates response to checkpoint inhibitor immunotherapy. Science.

[59] Qiu J., Chen L., Zhang L., Xu F., Zhang C., Ren G., Chang K., He G., Du Z., Le Y. (2023). Xie Zhuo Tiao Zhi formula modulates intestinal microbiota and liver purine metabolism to suppress hepatic steatosis and pyroptosis in NAFLD therapy. Phytomedicine.

[60] Alvarenga T.I.R.C., Chen Y., Furusho-Garcia I.F., Perez J.R.O., Hopkins D.L. (2015). Manipulation of omega-3 PUFAs in lamb: Phenotypic and genotypic views. Compr. Rev. Food Sci. Food Saf..

[61] Liu T., Zhang T.W., Zhang Y.N., Zhang M., Zhai M.Q., Wang W.H., Wang C.L., Duan Y., Jin Y. (2024). Exercise influences fatty acids in the longissimus dorsi muscle of Sunit lambs and improves dressing percentage by affecting digestion, absorption, and lipid metabolism. Qual. Assur. Saf. Crops Foods.

[62] Yu H., Yu S., Guo J., Wang J., Mei C., Raza S.H.A., Cheng G., Zan L. (2024). Comprehensive analysis of transcriptome and metabolome reveals regulatory mechanism of intramuscular fat content in beef cattle. J. Agric. Food Chem..

