# Supplementary material for: Integration of Serum and Liver Metabolomics with Antioxidant Biomarkers Elucidates Dietary Energy Modulation of the Fatty Acid Profile in Donkey Meat
Source: Antioxidants (Basel). 2026 Jan 21;15(1):140. doi: 10.3390/antiox15010140 (PMC12838122; doi:10.3390/antiox15010140)
Supplement: Supplementary file 1 [file antioxidants-15-00140-s001.zip › antioxidants-4067178-supplementary.pdf]

Table S1 Dietary composition in fattening period (air-dry basis, %)

Index	1-45d			46-90d			91-135d		
	LEG	MEG	HEG	LEG	MEG	HEG	LEG	MEG	HEG
Ingredient									
Millet straw	55.18	43.34	31.35	40.28	35.49	31.21	38.34	33.70	28.66
Alfalfa	2.12	11.84	21.42	4.02	6.03	7.99	2.04	4.06	6.08
Corn silage	6.43	9.77	12.16	6.09	9.13	11.34	5.55	8.30	11.51
Corn	6.03	14.81	20.25	27.12	30.45	29.73	37.43	36.52	35.73
Wheat middling	0.00	0.00	0.00	1.60	2.00	2.40	1.76	1.92	1.59
Soybean meal	8.40	8.00	5.60	7.20	6.40	5.60	6.22	3.96	1.21
Corn gluten meal	0.60	0.92	0.40	0.70	0.00	0.00	0.00	0.00	0.00
Corn germ meal	7.42	1.40	0.00	3.00	0.00	0.00	0.00	0.00	0.00
DDGS	1.60	3.40	3.00	3.16	3.76	3.00	3.47	2.20	3.02
Bran	10.00	3.90	0.00	3.10	0.00	0.00	0.00	0.00	0.00
Soybean oil	0.00	0.40	0.60	0.00	0.00	1.00	0.00	0.82	1.26
Puffed full-fat soybeans	0.00	0.00	3.00	1.00	4.00	5.00	2.20	5.50	7.92
NaCl	0.40	0.40	0.40	0.50	0.50	0.50	0.55	0.55	0.55
Limestone	0.44	0.44	0.44	0.56	0.56	0.56	0.61	0.61	0.61
CaHPO3	0.88	0.88	0.88	1.10	1.10	1.10	1.21	1.21	1.21
Premix[1]	0.20	0.20	0.20	0.20	0.20	0.20	0.20	0.20	0.20
NaHCO3	0.30	0.30	0.30	0.38	0.38	0.38	0.44	0.44	0.44
Total	100.00	100.00	100.00	100.00	100.00	100.00	100.00	100.00	100.00

Note: [1]Per g of premix provided the following: Fe 10 mg, Cu 5 mg, Zn 30 mg, Mn 30 mg, I 0.12 mg, Se 0.12 mg, Co 0.12 mg, VA 2 000 IU, VD3 1 200 IU, VE 9 IU, VK 120 mg, VB_1 0.24mg, VB_2 5. 7 mg, VB_6 0.6 mg, nicotinic acid 12 mg, D-pantothenic acid 11 mg, VB_{12} 0.36 mg, biotin 0.15 mg, folic acid 1. 50 mg.

Table S2 Fatty acid composition of the experimental diet (percentage of total fatty acid)

Fatty acids	1-45d			46-90d			91-135d		
	LEG	MEG	HEG	LEG	MEG	HEG	LEG	MEG	HEG
Saturated Fatty Acids									
C4:0	0.040	0.038	0.035	0.036	0.035	0.033	0.035	0.034	0.031
C6:0	0.032	0.027	0.022	0.025	0.024	0.022	0.024	0.024	0.024
C8:0	0.041	0.047	0.039	0.045	0.039	0.033	0.042	0.039	0.029
C10:0	0.117	0.106	0.073	0.096	0.090	0.078	0.090	0.080	0.071
C11:0	0.100	0.110	0.118	0.085	0.087	0.086	0.078	0.079	0.078
C12:0	0.926	0.779	0.624	0.691	0.635	0.585	0.651	0.598	0.541
C13:0	0.041	0.038	0.036	0.034	0.033	0.029	0.032	0.028	0.027
C14:0	0.863	0.784	0.704	0.664	0.633	0.610	0.617	0.596	0.563
C15:0	0.339	0.364	0.392	0.278	0.270	0.267	0.245	0.241	0.240
C16:0	21.414	21.875	22.640	19.863	20.425	20.223	19.053	19.918	19.782
C17:0	0.710	0.667	0.624	0.564	0.539	0.516	0.526	0.506	0.476
C18:0	4.128	4.155	4.083	3.739	3.927	4.116	3.624	4.023	4.147
C20:0	1.579	1.383	1.195	1.250	1.179	1.105	1.189	1.129	1.040
C21:0	0.224	0.200	0.179	0.172	0.160	0.152	0.162	0.151	0.140
C22:0	1.043	0.990	0.945	0.829	0.805	0.788	0.771	0.769	0.746
C23:0	0.626	0.726	0.817	0.528	0.532	0.532	0.465	0.468	0.469
C24:0	1.328	1.237	1.138	1.048	1.000	0.949	0.973	0.928	0.875
Monounsaturated fatty acids									
C14:1	0.048	0.030	0.028	0.043	0.026	0.026	0.022	0.024	0.042
C15:1	0.026	0.026	0.026	0.023	0.022	0.021	0.022	0.019	0.019
C17:1	0.081	0.074	0.074	0.068	0.067	0.071	0.065	0.068	0.070
C16:1	1.117	0.935	0.761	0.842	0.783	0.738	0.801	0.747	0.678
C18:1n9t	0.184	0.188	0.187	0.147	0.160	0.163	0.152	0.155	0.117
C18:1n9c	15.199	15.067	13.838	17.141	15.394	14.975	17.501	15.713	14.779
C20:1	0.296	0.235	0.246	0.219	0.221	0.273	0.214	0.266	0.295
C22:1	0.306	0.242	0.269	0.169	0.174	0.178	0.150	0.150	0.162
C24:1	0.072	0.069	0.065	0.056	0.056	0.054	0.053	0.050	0.051
n-6 Polyunsaturated Fatty Acids									
C18:2n6t	0.027	0.033	0.037	0.028	0.030	0.033	0.026	0.029	0.037
C18:2n6c	34.780	35.203	35.769	39.311	39.679	39.658	40.801	40.253	40.804

C18:3n6	0.138	0.160	0.176	0.113	0.112	0.162	0.105	0.136	0.159
C20:2n6	0.101	0.138	0.186	0.091	0.106	0.116	0.085	0.108	0.118
C20:3n6	0.028	0.024	0.032	0.024	0.027	0.026	0.029	0.038	0.035
C20:4n6	0.651	0.636	0.620	0.504	0.478	0.456	0.463	0.437	0.405
C22:2n6	0.062	0.063	0.072	0.045	0.046	0.047	0.042	0.044	0.044
n-3 Polyunsaturated Fatty Acids									
C18:3n3	12.756	12.742	13.325	10.761	11.710	12.399	10.423	11.675	12.464
C20:3n3	0.107	0.156	0.209	0.106	0.117	0.123	0.093	0.099	0.101
C20:5n3	0.329	0.378	0.418	0.277	0.277	0.281	0.259	0.271	0.268
C22:6n3	0.158	0.139	0.124	0.120	0.123	0.106	0.117	0.117	0.104
Sum and Ratio[1]									
SFA	33.552	33.524	33.662	29.946	30.413	30.123	28.577	29.612	29.280
MUFA	17.329	16.867	15.494	18.708	16.903	16.499	18.980	17.193	16.213
PUFA	49.136	49.671	50.968	51.379	52.706	53.407	52.445	53.205	54.539
n-3PUFA	13.350	13.414	14.076	11.264	12.228	12.910	10.893	12.161	12.937
n-6PUFA	35.786	36.257	36.892	40.115	40.478	40.498	41.552	41.044	41.602
n-3LCPUFA	0.594	0.672	0.751	0.503	0.517	0.511	0.470	0.486	0.473
n-6LCPUFA	0.841	0.860	0.910	0.664	0.657	0.645	0.619	0.627	0.601
n-6/n-3	6.497	6.568	4.370	8.517	5.881	4.794	8.070	5.275	4.514
U/S	2.538	2.590	2.564	3.059	2.844	2.867	3.274	2.897	2.914
P/S	1.846	1.890	1.931	2.207	2.138	2.177	2.374	2.178	2.241

1SFA= saturated fatty acid; MUFA=monounsaturated fatty acids; PUFA= polyunsaturated fatty acids; LCPUFA =long chain polyunsaturated fatty acids; n-6/n-3 = n-6 PUFA/n-3 PUFA; U/S=unsaturated fatty acid/SFA; P/S = PUFA/SFA.

Table S3 Effects of dietary energy level on the fatty acid composition of longissimus thoracis muscle of meat donkeys (g/100g total fatty acid)

Fatty acids	LEG	MEG	HEG	SEM	*P*-value
SFA					
C4:0	0.035	0.031	0.026	0.006	0.497
C6:0	0.010[b]	0.015[b]	0.028[a]	0.004	0.021
C8:0	0.012	0.011	0.009	0.002	0.665
C10:0	0.042	0.035	0.038	0.003	0.309
C11:0	0.019	0.012	0.011	0.008	0.462
C12:0	0.126	0.1	0.116	0.009	0.125
C13:0	0.01	0.012	0.015	0.003	0.795
C14:0	1.805	1.657	1.758	0.108	0.619
C15:0	0.146	0.148	0.132	0.009	0.743
C16:0	25.254	24.816	24.453	0.366	0.321
C17:0	0.277[a]	0.237[b]	0.223[b]	0.013	0.018
C18:0	6.523[b]	7.521[a]	6.962[ab]	0.262	0.044
C20:0	0.064	0.075	0.07	0.003	0.07
C21:0	0.035	0.035	0.033	0.003	0.875
C22:0	0.013	0.019	0.014	0.003	0.817
C23:0	2.2	2.602	2.608	0.162	0.149
C24:0	0.015[b]	0.025[a]	0.017[b]	0.001	<0.001
MUFA					
C14:1	0.179	0.17	0.209	0.014	0.151
C15:1	0.052[a]	0.038[b]	0.039[b]	0.004	0.023
C16:1	4.349	4.666	5.228	0.258	0.073
C17:1	0.065	0.062	0.067	0.005	0.784
C20:1	0.277[ab]	0.265[b]	0.296[a]	0.007	0.013
C22:1	0.026	0.031	0.032	0.003	0.317
C24:1	0.008[ab]	0.009[a]	0.007[b]	0.001	0.035
n-6PUFA					
C20:2n6	0.552	0.557	0.575	0.027	0.81
C20:3n6	0.28	0.919	0.284	0.022	0.919
C20:4n6	0.011	0.02	0.016	0.003	0.078
n-3PUFA					
C22:6n3	0.096	0.103	0.084	0.012	0.81
Sum and Ratio[1]					
SFA	36.437	37.296	36.227	0.564	0.382
MUFA	34.887	35.893	33.947	0.887	0.32
n-3LCPUFA	0.283	0.385	0.377	0.043	0.194
n-6LCPUFA	0.877	0.953	0.929	0.052	0.582

SFA= saturated fatty acid; MUFA=monounsaturated fatty acids; PUFA= polyunsaturated fatty acids; LCPUFA =long chain polyunsaturated fatty acids; n-6/n-3 = n-6 PUFA/n-3 PUFA; U/S=unsaturated fatty acid/SFA; P/S = polyunsaturated fatty acids/saturated fatty acids; LEG = low-energy group. MEG= medium energy group. HEG = high-energy group. SEM = standard error of least square means. [abc] At $P < 0.05$, means in the same row that are followed by the same superscript letters do not differ substantially.

Table S4 Effects of dietary energy level on the fatty acid composition of subcutaneous adipose tissue of meat donkeys (g/100g total fatty acid)

Fatty acids	LEG	MEG	HEG	SEM	*P*-value
SFA					
C4:0	0.019[a]	0.007[b]	0.006[b]	0.001	<0.001
C6:0	0.003	0.004	0.004	0.001	0.594
C8:0	0.005[a]	0.004[b]	0.004[b]	0.0001	0.046
C10:0	0.06	0.053	0.064	0.006	0.445
C11:0	0.003	0.003	0.004	0.001	0.348
C12:0	0.181	0.188	0.2	0.018	0.754
C13:0	0.008	0.007	0.006	0.001	0.052
C14:0	2.384	2.347	2.204	0.119	0.54
C15:0	0.208	0.17	0.183	0.012	0.103
C16:0	25.687[a]	24.112[b]	24.263[b]	0.235	<0.001
C17:0	0.317	0.254	0.276	0.018	0.106
C18:0	4.115	3.852	4.097	0.233	0.679
C20:0	0.064	0.062	0.07	0.004	0.326
C21:0	0.059	0.05	0.051	0.005	0.394
C22:0	0.009	0.011	0.011	0.001	0.422
C23:0	0.006[c]	0.085[b]	0.097[a]	0.004	<0.001
C24:0	0.003[b]	0.004[a]	0.004[ab]	0.0001	0.037
MUFA					
C14:1	0.224	0.239	0.18	0.021	0.142
C15:1	0.005	0.005	0.004	0.001	0.605
C16:1	5.181	5.343	4.68	0.348	0.427
C17:1	0.347[a]	0.293[b]	0.275[b]	0.015	0.008
C20:1	0.385[ab]	0.367[b]	0.415[a]	0.011	0.026
C22:1	0.027	0.024	0.024	0.002	0.565
C24:1	0.005[b]	0.004[b]	0.006[a]	0.0001	0.048
n-6PUFA					
C20:2n6	0.612[a]	0.512[b]	0.595[a]	0.026	0.026
C20:3n6	0.052	0.051	0.055	0.064	0.416
C20:4n6	0.118	0.126	0.107	0.019	0.441
n-3PUFA					
C22:6n3	0.018	0.016	0.017	0.001	0.668
Sum and Ratio[1]					
SFA	32.259	31.403	31.645	0.337	0.203
MUFA	38.379[a]	38.943[a]	34.549[b]	0.444	<.0001
n-3LCPUFA	0.117	0.104	0.088	0.01	0.332
n-6LCPUFA	0.807[a]	0.670[b]	0.810[a]	0.03	0.004

SFA= saturated fatty acid; MUFA=monounsaturated fatty acids; PUFA= polyunsaturated fatty acids; LCPUFA =long chain polyunsaturated fatty acids; n-6/n-3 = n-6 PUFA/n-3 PUFA; U/S=unsaturated fatty acid/SFA; P/S = polyunsaturated fatty acids/saturated fatty acids; LEG = low-energy group. MEG= medium energy group. HEG = high-energy group. SEM = standard error of least square means. [abc] At $P < 0.05$, means in the same row that are followed by the same superscript letters do not differ substantially.

Table S5 Effects of dietary energy level on serum fatty acid composition of meat donkeys (g/100g total fatty acid)

Fatty acids	LEG	MEG	HEG	SEM	*P*-value
SFA					
C4:0	0.142	0.142	0.103	0.018	0.245
C6:0	0.096^{a}	0.103^{a}	0.069^{b}	0.009	0.024
C8:0	0.062^{a}	0.040^{b}	0.035^{b}	0.006	0.019
C10:0	0.085	0.103	0.098	0.012	0.371
C11:0	0.071^{b}	0.130^{a}	0.118^{a}	0.008	0.001
C12:0	0.162^{a}	0.097^{b}	0.151^{a}	0.015	0.013
C13:0	0.149	0.144	0.149	0.015	0.898
C14:0	0.526^{a}	0.497^{a}	0.405^{b}	0.027	0.013
C15:0	0.24	0.204	0.197	0.018	0.208
C16:0	18.177^{a}	16.730^{ab}	15.704^{b}	0.493	0.007
C17:0	0.665	0.612	0.684	0.056	0.652
C18:0	20.597	18.124	18.871	1.04	0.249
C20:0	0.627^{b}	0.738^{a}	0.788^{a}	0.028	0.002
C21:0	0.117^{a}	0.047^{b}	0.033^{b}	0.018	0.006
C22:0	0.353^{b}	0.358^{b}	0.438^{a}	0.016	0.002
C23:0	0.057	0.044	0.038	0.007	0.336
C24:0	0.052^{b}	0.040^{ab}	0.062^{a}	0.005	0.019
MUFA					
C14:1	0.170^{a}	0.088^{b}	0.123^{b}	0.013	0.001
C15:1	0.094^{b}	0.137^{a}	0.146^{a}	0.014	0.035
C16:1	0.952^{a}	0.866^{ab}	0.657^{b}	0.072	0.024
C17:1	0.012^{b}	0.040^{a}	0.047^{a}	0.003	0.001
C20:1	0.403	0.396	0.381	0.016	0.833
C22:1	5.417	4.876	5.205	0.228	0.263
C24:1	0.232^{b}	0.283^{b}	0.376^{a}	0.028	0.006
n-6PUFA					
C20:2n6	0.838	0.808	0.788	0.067	0.868
C20:3n6	0.205^{b}	0.266^{a}	0.258^{a}	0.011	0.002
C20:4n6	0.858	0.762	0.823	0.04	0.251
n-3PUFA					
C22:6n3	0.077	0.078	0.07	0.011	0.978
Sum and Ratio[1]					
SFA	39.882	38.075	38.446	0.798	0.262
MUFA	21.498^{a}	19.331^{b}	18.970^{b}	0.284	<0.001
n-3LCPUFA	0.34	0.372	0.395	0.029	0.433
n-6LCPUFA	2.128	2.044	2.09	0.039	0.272

SFA= saturated fatty acid; MUFA=monounsaturated fatty acids; PUFA= polyunsaturated fatty acids; LCPUFA =long chain polyunsaturated fatty acids; n-6/n-3 = n-6 PUFA/n-3 PUFA; U/S=unsaturated fatty acid/SFA; P/S = polyunsaturated fatty acids/saturated fatty acids; LEG = low-energy group. MEG= medium energy group. HEG = high-energy group. SEM = standard error of least square means. [abc] At $P < 0.05$, means in the same row that are followed by the same superscript letters do not differ substantially.

Table S6 Effects of dietary energy level on fatty acid composition in liver of meat donkeys (g/100g total fatty acid)

Fatty acids	LEG	MEG	HEG	SEM	*P*-value
SFA					
C4:0	0.021^{a}	0.016^{b}	0.021^{a}	0.001	0.002
C6:0	0.013	0.016	0.017	0.002	0.381
C8:0	0.010^{ab}	0.007^{b}	0.013^{a}	0.001	0.008
C10:0	0.009	0.009	0.008	0.001	0.920
C11:0	0.007	0.004	0.006	0.001	0.263
C12:0	0.019	0.021	0.019	0.002	0.586
C13:0	0.015^{a}	0.004^{b}	0.008^{b}	0.002	0.001
C14:0	0.296	0.32	0.315	0.015	0.505
C15:0	0.137	0.115	0.122	0.008	0.149
C16:0	10.884	10.307	10.648	0.491	0.710
C17:0	0.5	0.445	0.502	0.026	0.238
C18:0	27.137	25.853	24.549	0.838	0.117
C20:0	0.241^{b}	0.267^{ab}	0.310^{a}	0.018	0.042
C21:0	0.027	0.03	0.03	0.003	0.718
C22:0	0.040^{c}	0.059^{b}	0.078^{a}	0.005	<0.001
C23:0	3.312^{ab}	3.002^{b}	3.752^{a}	0.198	0.045
C24:0	0.044^{b}	0.045^{b}	0.056^{a}	0.01	0.029
MUFA					
C14:1	0.039^{a}	0.013^{b}	0.043^{a}	0.003	0.005
C15:1	0.022	0.02	0.02	0.002	0.473
C16:1	0.908	1.073	1.126	0.092	0.357
C17:1	0.077	0.163	0.183	0.036	0.394
C20:1	0.447^{b}	0.504^{b}	0.634^{a}	0.029	0.001
C22:1	0.041	0.047	0.054	0.005	0.375
C24:1	0.028^{b}	0.032^{ab}	0.038^{a}	0.002	0.02
n-6PUFA					
C20:2n6	1.439^{b}	1.426^{b}	1.794^{a}	0.098	0.023
C20:3n6	0.468	0.442	0.486	0.026	0.498
C20:4n6	0.018^{ab}	0.015^{b}	0.024^{a}	0.002	0.022
n-3PUFA					
C22:6n3	0.046	0.044	0.047	0.004	0.884
Sum and Ratio[1]					
SFA	42.99^{a}	40.96^{b}	38.15^{c}	0.559	0.001
MUFA	13.29	14.1	14.19	0.676	0.591
n-3LCPUFA	0.16^{b}	0.19^{ab}	0.22^{a}	0.013	0.018
n-6LCPUFA	1.911^{b}	1.961^{b}	2.37^{a}	0.129	0.039

SFA= saturated fatty acid; MUFA=monounsaturated fatty acids; PUFA= polyunsaturated fatty acids; LCPUFA =long chain polyunsaturated fatty acids; n-6/n-3 = n-6 PUFA/n-3 PUFA; U/S=unsaturated fatty acid/SFA; P/S = polyunsaturated fatty acids/saturated fatty acids; LEG = low-energy group. MEG= medium energy group. HEG = high-energy group. SEM = standard error of least square means. ^{abc} At $P < 0.05$, means in the same row that are followed by the same superscript letters do not differ substantially.

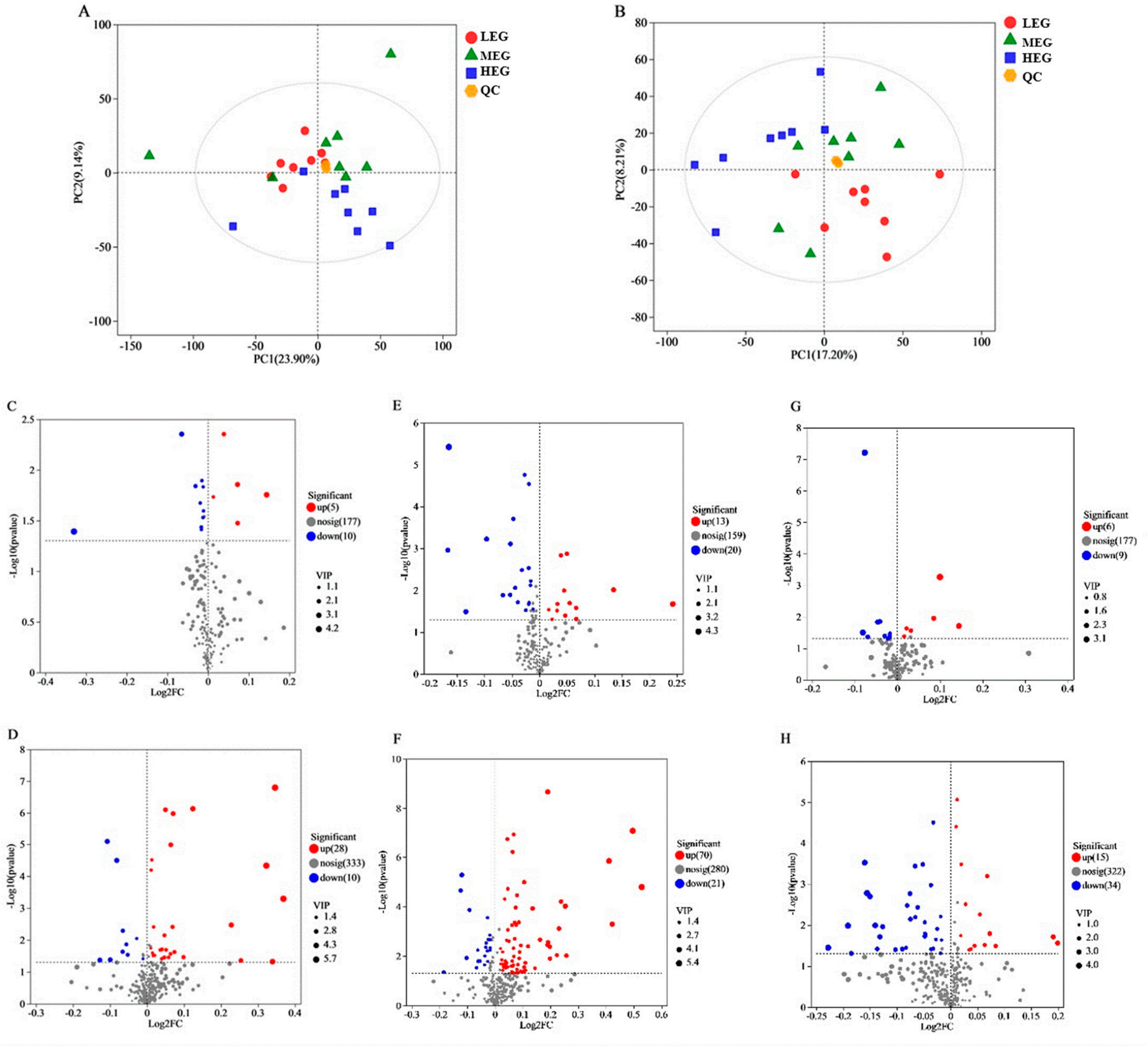

Figure S1. Serum metabolome profiles between MEG vs LEG, HEG vs LEG, MEG vs HEG. (A) Principal component analysis (PCA) in Pos. (B) PCA in Neg. (C) Differential metabolites in MEG vs LEG in Pos. (D) Differential metabolites in MEG vs LEG in Neg. (E) Differential metabolites in HEG vs LEG in Pos. (F) Differential metabolites in HEG vs LEG in Neg. (G) Differential metabolites in MEG vs HEG in Pos. (H) Differential metabolites in MEG vs HEG in Neg. The red curves represent upregulated metabolites, the blue represents downregulated metabolites, and the gray represents no change. LEG = low-energy group. MEG= medium energy group. HEG = high-energy group.

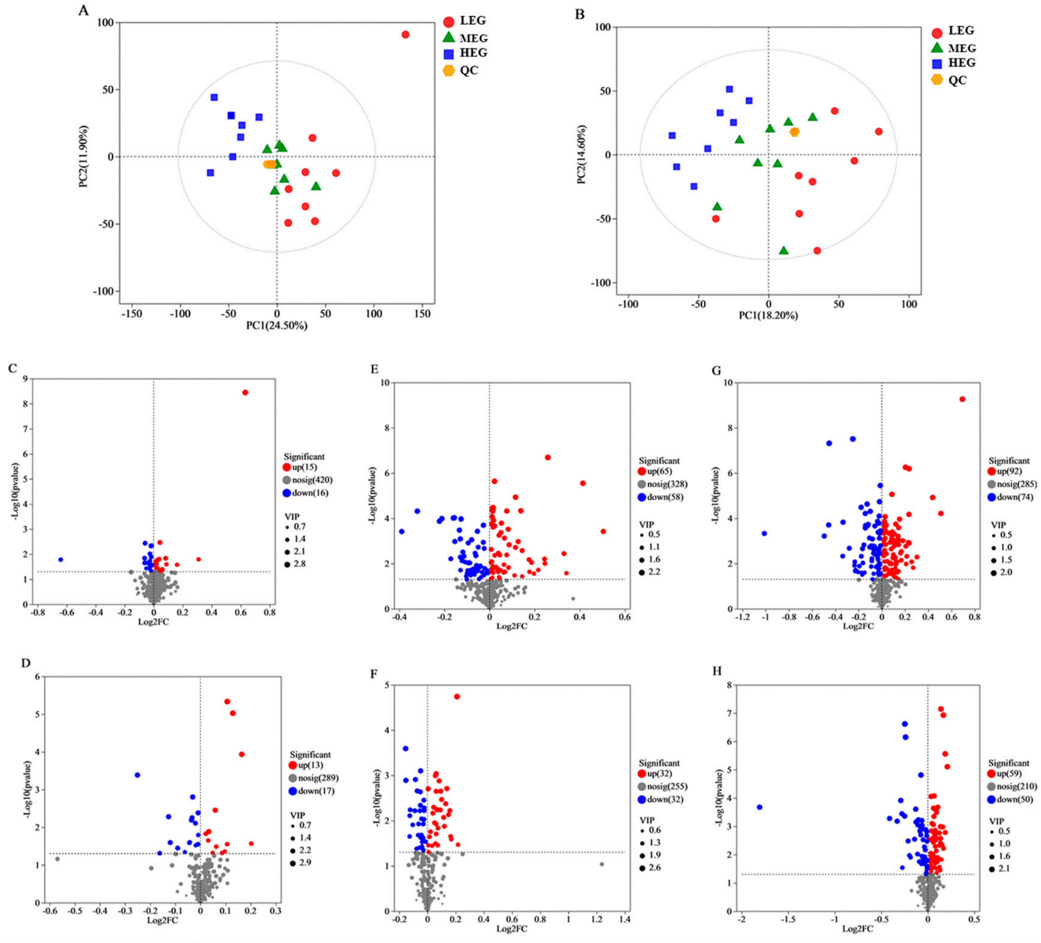

Figure S2. Liver metabolome profiles between MEG vs LEG, HEG vs LEG, MEG vs HEG. (A) Principal component analysis (PCA) in Pos. (B) PCA in Neg. (C) Differential metabolites in MEG vs LEG in Pos. (D) Differential metabolites in MEG vs LEG in Neg. (E) Differential metabolites in HEG vs LEG in Pos. (F) Differential metabolites in HEG vs LEG in Neg. (G) Differential metabolites in MEG vs HEG in Pos. (H) Differential metabolites in MEG vs HEG in Neg. The red curves represent upregulated metabolites, the blue represents downregulated metabolites, and the gray represents no change. LEG = low-energy group. MEG= medium energy group. HEG = high-energy group.

Table S7 Primer pairs sequences for quantitative real-time PCR

gene	Primer sequences (5′-3′)[1]	Gen Bank no.	Annealing temperature （℃）	length （bp）
GAPDH	F: GTGGAGGGACTCATGACCAC	XM_014834961.1	60℃	173
	R: AGCTTCCCATTCAGCTCAGG			
ACTB	F: CGGCGGCGCCCTATAAAA	XM_023655002.1	60℃	140
	R: CGTCATCCATGGCTAGCTGGG			
ACACA	F: TACCTTCTTCTACTGGCGGCTGAG	XM_014862267.1	60℃	140
	R: CTGTCCCTTCCACTTCCACAAACC			
ELOVL2	F: AGAAATATCTCACGCAGGCTCAGC	XM_014844387.1	60℃	80
	R: ACACGGTTTCACGACAGCACTC			
SCD	F: AGACATCCGCCCTGAAATGA	XM_014865738.1	60℃	192
	R: TAGTAGAAAGCCACCCAGAGG			
FASN	F: CTTCGACAACCGAGATAGCC	XM_023651723.1	60℃	121
	R: GTGAGGGCCGTAGTCATAGC			
LPL	F: AGCGCTCCATTCATCTCTTC	XM_014841275.1	60℃	123
	R: AACGGTTCTTTCTGCAGCTC			
PPARγ	F: AGGAGAAGCTGTTGGCAGAG	XM_014838392.1	60℃	119
	R: GGTCAGTGGGAAGGACTTGA			
ELOVL5	F: ACGCCACCATGCTGAACATCTG	XM_014863868.1	62℃	134
	R: GGAGGGGATGGACGACAGACC			
FADS1	F: CTGGCTCACTCTTTGGGTCTTTGG	XM_014866888.1	64℃	150
	R: AGCAGGTGGTTCCACGTAGAGG			
LIPE	F: AGAGGAGATGAGCACGAAGGACAG	XM_014867527.1	64℃	122
	R: TGGCGATGGGCGACGAGTAG			

Note: F=Forward primer; R=Reverse primer.
